# Interplay of a non-conjugative integrative element and a conjugative plasmid in the spread of antibiotic resistance via suicidal plasmid transfer from an aquaculture *Vibrio* isolate

**DOI:** 10.1371/journal.pone.0198613

**Published:** 2018-06-07

**Authors:** Lisa Nonaka, Tatsuya Yamamoto, Fumito Maruyama, Yuu Hirose, Yuki Onishi, Takeshi Kobayashi, Satoru Suzuki, Nobuhiko Nomura, Michiaki Masuda, Hirokazu Yano

**Affiliations:** 1 Department of Microbiology, Dokkyo Medical University School of Medicine, Mibu, Tochigi, Japan; 2 Faculty of Life and Environmental Sciences, University of Tsukuba, Tennodai, Tsukuba, Japan; 3 Graduate School of Medicine, Kyoto University, Kyoto, Japan; 4 Department of Environmental and Life Sciences, Toyohashi University of Technology, Tempaku, Toyohashi, Aichi, Japan; 5 Center for Marine Environmental Studies, Ehime University, Matsuyama, Ehime, Japan; 6 Graduate School of Medicine, Ehime University, To-on, Ehime, Japan; Institut National de la Recherche Agronomique, FRANCE

## Abstract

The capture of antimicrobial resistance genes (ARGs) by mobile genetic elements (MGEs) plays a critical role in resistance acquisition for human-associated bacteria. Although aquaculture environments are recognized as important reservoirs of ARGs, intra- and intercellular mobility of MGEs discovered in marine organisms is poorly characterized. Here, we show a new pattern of interspecies ARGs transfer involving a ‘non-conjugative’ integrative element. To identify active MGEs in a *Vibrio ponticus* isolate, we conducted whole-genome sequencing of a transconjugant obtained by mating between *Escherichia coli* and *Vibrio ponticus*. This revealed integration of a plasmid (designated pSEA1) into the chromosome, consisting of a self-transmissible plasmid backbone of the MOB_H_ group, ARGs, and a 13.8-kb integrative element Tn*6283*. Molecular genetics analysis suggested a two-step gene transfer model. First, Tn*6283* integrates into the recipient chromosome during suicidal plasmid transfer, followed by homologous recombination between the Tn*6283* copy in the chromosome and that in the newly transferred pSEA1. Tn*6283* is unusual among integrative elements in that it apparently does not encode transfer function and its excision barely generates unoccupied donor sites. Thus, its movement is analogous to the transposition of insertion sequences rather than to that of canonical integrative and conjugative elements. Overall, this study reveals the presence of a previously unrecognized type of MGE in a marine organism, highlighting diversity in the mode of interspecies gene transfer.

## Introduction

Aquaculture environments are now noticed to have an important impact on human health by contributing to the emergence of antibiotic resistant strains [1]. Similar to other environments, the ARGs found in aquatic environments are frequently accompanied by mobile genetic elements (MGEs) such as conjugative plasmids, integrative and conjugative elements (ICEs), transposons, and integrons [2–5]. Thus, the capture of ARGs by MGEs is considered to play a critical role in introducing ARGs to human-associated pathogens from an environmental reservoir [6]. Furthermore, since identical ARGs have been discovered in different bacterial species obtained from the same aquaculture site, they are assumed to be transmitted by horizontal gene transfer (HGT) [7, 8, 9]. In particular, conjugative transferable elements, including A/C plasmids [10], pAQU1-like plasmids [4, 11], and SXT/R391-like ICEs [5, 12, 13], all belonging to the MOB_H_ group of conjugative elements [14], have frequently been identified in members of *Gammaproteobacteria* such as *Enterobacteriaceae* and *Vibrionaceae* isolated from aquatic environments. However, novel types of plasmids also most likely exist in marine isolates [4]. Thus, aquatic environments might preserve diverse and distinct MGEs and HGT mechanisms. Identification of this diversity is important for understanding the mechanisms and key players of HGT in the environment, which can help to inform the development of effective strategies for controlling the emergence and dissemination of ARGs in aquaculture environments.

Previously, we isolated *Vibrio ponticus* strain 04Ya108 from an aquaculture site in Japan, which showed a multidrug-resistance phenotype [7]. This strain did not possess known transfer operon (*tra*) genes based on PCR detection, but could nevertheless transfer some known ARGs to an *E*. *coli* laboratory strain through mating, which integrated in the chromosome of transconjugants [4]. This finding suggested the presence of an MGE in strain 04Ya108 that mediates interspecies gene transfer.

To test this hypothesis, in this study, we sought to identify relevant MGEs in the *E*. *coli* transconjugant by conducting genome sequencing. We further characterized the MGE and determined its relationship to known MGEs. This revealed a novel ‘non-conjugative’ integrative element embedded in a plasmid. To date, integrative elements without mobilization function have not been well studied, and little information is available for their excision and integration pattern or survival strategy as genetic parasites. Thus, we conducted further experiments to obtain this basic information by determining the mobility unit of the MGE and sequenced the expected recombination joint formed upon its excision. Further, the effects of the non-conjugative integrative element on growth and fitness in the *E*. *coli* host were evaluated. The results of this study can help to shed light on the diversity in the mode of interspecies gene transfer and highlight the potential of the sea environment as an important reservoir of unrecognized types of MGEs.

## Materials and methods

### Bacterial strains and culture media

*Vibrio ponticus* strain 04Ya108 was isolated from the sediment of a coastal aquaculture site (latitude, 34.378499; longitude, 134.103576) near Yashima, Kagawa prefecture, Japan [7]. This strain was cultured at 25°C in a modified brain heart infusion medium (BD Biosciences, San Jose, CA, USA) containing 2.5% NaCl and 10 μg/ml of tetracycline (Nacalai Tesque, Kyoto, Japan). The *E*. *coli* strains used were W3110, W3110Rif^r^ [11], BW25113, JW2669 (BW25113 Δ*recA*::*kan*), TJ108W0 (W3110 *bcp*::Tn*6283*::pSEA1, carrying three copies of Tn*6283*), LN3 (W3110 Δ*bcp*::FRT), LN5 (W3110 *bcp*::Tn*6283*, one copy of Tn*6283*), and LN7 (W3110 *bcp*::Tn*6283*::pSEA1, two copies of Tn*6283*). W3110, BW25113 [15], and JW2669 [15] were obtained from the National Bio Resource Project (National Institute of Genetics, Japan). Strain LN3 was constructed by removing the *bcp* gene from strain W3110 using lambda-Red recombinase expressed from pKD46 and the PCR-amplified chloramphenicol resistance gene (*cat*) cassette of pKD3 [16] using the primer set LN054-LN055 (Table 1). The *cat* gene in the LN3 parental strain was removed from the chromosome using pFLP3 [17]. TJ108W0 is an *E*. *coli* transconjugant obtained by mating of strain W3110 and strain 04Ya108, with subsequent selection for growth at 42°C and tetracycline resistance [11]. Strain LN5 is one of the randomly selected tetracycline-sensitive clones derived from transconjugant LN7, which initially carried two copies of Tn*6283* in the chromosome. Strain LN5 was obtained by conducting three cycles of serial batch-culture transfer of LN7 in LB at 37°C. In one cycle, 3 μl of 12-hr-old culture was transferred into 3 ml of fresh medium. The absence of the pSEA1 backbone in strain LN5 was confirmed by PCR [4]. *E*. *coli* strains were cultured at 37°C in LB medium (10 g tryptone, 5 g yeast extract, 10 g NaCl per liter of water; BD Bioscience, San Jose, CA, USA). Antibiotics were added to the medium at the following concentrations: tetracycline, 10 μg/ml for liquid media and 20 μg/ml for solid media; kanamycin, 25 μg/ml; rifampicin, 100 μg/ml.

**Table 1:** Primers used in this study.

Primer name	Primer sequence (5' to 3')	Target gene or region(s); purpose	Reference
3F ^a^	CCAAAAGTGAGCTGGGTGGCAAT	Primer set 3, 6 in S4 Fig; *att*_Tn*6283*_ in Fig 4 and S5 Fig; integration pattern analysis, joint sequencing	This study
3R ^a^	GCGTCACTTCTCCAGTGTCGATA	Primer set 2, 5 in S4 Fig; *att*_Tn*6283*_ in Fig 4 and S5 Fig; integration pattern analysis	This study
2F ^a^	CTCGGTTGACGTTGTTGGTTTGC	Primer set 4, 5 in S4 Fig; integration pattern analysis, joint sequencing	This study
2F2 ^a^	CAATGCCCCCTTTTCACTTA	Primer set 1, 3 in S4 Fig; Scar1/Scar2 in S5 Fig; integration pattern analysis, joint sequencing	This study
1R3 ^b^	CGGTCAATATGGCACCTTTT	Scar1/Scar2 in S5 Fig; joint sequencing	This study
2599370F ^a^	CCGTTTTGCGGAAAAAGAGCTGC	Primer set 1, 2, 7 in S4 Fig, attB*/ Scar in Fig 4; integration pattern analysis, joint sequencing, qPCR	This study
2599572R ^a^	CGTCGTGGTGATTGCTGGTTTTG	Primer set 4, 6, 7 in S4 Fig, attB*/ Scar in Fig 4; integration pattern analysis, qPCR	This study
LN005	CTCAACCTTTGCAACCCATT	*intA* [pSEA1_001]; qPCR, probe	This study
LN006	TTGAGTGCCCCATTAACTCC	*intA* [pSEA1_001]; qPCR, probe	This study
LN009	CGATAGAGGGAGCTGACGAG	*traI* [pSEA1_097]; qPCR	This study
LN010	CGGTTCAGTTCCAGGTTGTT	*traI* [pSEA1_097]; qPCR	This study
LN011	TGACAACTGCTATGCTTGAGATT	*att*_Tn*6283*_; qPCR	This study
LN012	ACCCTCGATATGTTAGCCACA	*att*_Tn*6283*_; qPCR	This study
LN015	TCAAACGATGGCGTCTATGCT	Scar1/ Scar2, *att*_pSEA1_; qPCR	This study
LN016	TCGTCCTCACCTGAATCCTGC	Scar1/ Scar2, *att*_pSEA1_; qPCR	This study
LN054	ATAAGAAAACTCATTTCAGAGTAAATTAAAGAAAGTAAGGATAATCCATGTGTAGGCTGGAGCTGCTTCG	*bcp*; lambda-RED recombination in *E*. *coli*	This study
LN055	TTGACTTTACCTCTTACCAGTTGGGCTGTGTTAGATTTTAATTCGGTTTACATATGAATATCCTCCTTA	*bcp*; lambda-RED recombination in *E*. *coli*	This study
dxs-F	CGAGAAACTGGCGATCCTTA	*dxs*; qPCR	[18]
dxs-R	CTTCATCAAGCGGTTTCACA	*dxs*; qPCR	[18]
tet(m)-1	GTTAAATAGTGTTCTTGGAG	*tet*(M) [pSEA1_229]; probe	[7]
tet(m)-2	CTAAGATATGGCTCTAACAA	*tet*(M) [pSEA1_229]; probe	[7]

^a^Primers used in primer sets 1 to 7, and their respective PCR product size are shown in S4 Fig.

^b^1R3 anneals to the 39-bp upstream region of the 2F annealing region.

### General DNA manipulations

Genomic DNA was extracted using the QuickGene DNA tissue kit (KURABO INDUSTRIES LTD., Osaka, Japan). Conventional PCR was performed using Taq polymerase (New England Biolabs, Ipswich, MA, USA). Long PCR was performed as follows. The PCR mixture (total volume, 10 μl) contained 20 pmol each of the primers, 1 μl of 10x LA PCR buffer II (Takara Bio Inc., Kusatsu, Japan), 4 mM each dNTP, 25 mM of MgCl_2_, 0.5 U of LA Taq DNA polymerase (Takara Bio Inc.), and template DNA (20–50 ng). Two-step PCR was performed with 30 cycles of denaturation at 96°C for 20 sec and extension at 69°C for 16 min. When necessary, PCR products were cloned into pGEM-T easy vector (Promega Corp., Madison, WI, USA) using Competent High DH5α competent cells (TOYOBO CO., LTD., Osaka, Japan). Sanger sequencing was performed using BigDye v 3.1 (Thermo Fisher Scientific, Waltham, MA, USA) and ABI 3130xl Genetic Analyzer (Thermo Fisher Scientific).

### Genome sequencing of a transconjugant

The transconjugant TJ108W0 was previously obtained by conjugation using strain 04Ya108 and strain W3110 as the donor and recipient, respectively [4]. The short reads for the TJ108W0 genome were obtained by pyrosequencing using the Genome Sequence FLX454+ platform. Two types of libraries were constructed and sequenced separately. One was an approximately 800-bp-long library designed to obtain up to 700-bp-long single-end reads. The other was an approximately 8-kb-long library designed to obtain up to 400-bp paired-end reads.

We conducted read assembly twice to identify MGEs in the transconjugant genome. First, 700-bp reads (225,251 reads) were mapped to the W3110 genome. The unmapped reads were then collected and assembled *de novo* using the GS assembler (Newbler 2.8). This yielded eight contigs potentially derived from MGEs of *V*. *ponticus*. Next, the ~400-bp paired reads (271,612 reads) and ~700-bp reads were assembled together. This yielded 82 scaffolds for a total of 151 contigs. One scaffold containing an MGE was detected by conducting a homology search against scaffolds obtained in the second assembly using the eight contigs obtained from the first assembly as queries. Gaps in the scaffold containing the MGE were filled by sequencing of the PCR products amplified with primers corresponding to the sequences close to the terminal ends of each contig using Applied Biosystems 3130xl Genetic Analyzer. The integration site of pSEA1 in the chromosome was identified as described in the Results section. The exact integration position was determined by PCR with primers 2599370F and 2599572R that anneal to the sequences in the flanking regions of the estimated target site (Table 1). Tn*6283*-flanking regions on pSEA1 in strain 04Ya108 was determined by PCR amplification of the Tn*6283*-pSEA1 backbone borders and subsequent direct sequencing of the PCR products. Annotation for the protein-coding regions in pSEA1 was first carried out using MetaGeneAnnotator [19] and then manually curated as described previously [11]. The circular plasmid map was drawn using In Silico Molecular Cloning Genomics Edition (In Silico Biology, Yokohama, Japan). Annotation for pSEA1 is shown in S1 Dataset.

### Conjugation experiments

Filter mating was performed as previously described [11]. Mating of *V*. *ponticus* 04Ya108 with *E*. *coli* strains was performed at 25°C on a 0.45-μm pore size nitrocellulose filter (Merck Millipore Ltd. Co., Cork, Ireland) placed on Marine agar plates (BD Bioscience, San Jose, CA USA). *E*. *coli* transconjugants were selected on LB agar plates with 20 μg/ml of tetracycline at 42°C, which is the temperature that inhibits growth of the donor strain. We obtained 20 *E*. *coli* transconjugants from independent filter mating of *V*. *ponticus* 04Ya108 and *E*. *coli* W3110, designated as strains LN7 to LN26.

The transferability of pSEA1 from the *E*. *coli* transconjugants to a secondary *E*. *coli* recipient was tested using the rifampicin-resistant strain W3110Rif^r^ as the recipient strain. The transconjugants were selected as previously described [4]. The transfer frequencies are expressed as CFU of the transconjugants per CFU of the donors.

### Pulsed-field gel electrophoresis (PFGE) and Southern hybridization

To investigate whether the integration of pSEA1 into the *bcp* gene in the *E*. *coli* chromosome is reproducible, genomes of the 20 newly obtained transconjugants were separated by PFGE and the position of the *tet(M)* gene was analyzed by Southern hybridization. Cells from 1-ml overnight culture of transconjugants (BW25113 derivatives) were collected in 1.5-ml tubes and respectively embedded in plugs, and then the DNA was separated using PFGE as previously described [4]. The probe was prepared by PCR amplification of *tet(M)* with the primer set tet(m)-1/ tet(m)-2, and detected using DIG DNA Labeling and Detection Kit for color metric detection (Roche, Basel, Switzerland). The fragment size was inferred based on the position of ProMega-Markers Lambda Ladders (Promega, Madison, Wisconsin) in the ethidium bromide stained gel. Additional Southern hybridization experiments were performed to investigate variation of the Tn*6283* integration pattern in the chromosome. Genomic DNA (2.5 μg) from each strain was digested with *Eco*NI and *Eco*RI or *Sac*I (New England Biolabs, Ipswich, MA, USA), separated on 0.8% SeaKem Gold agarose gels (Lonza Rockland, ME, USA), and transferred to Hybond-N^+^ using standard methods. The position of Tn*6238* DNA was determined by Southern hybridization using *intA* as a probe, which was obtained using PCR DIG Synthesis Kit (Roche, Basel, Switzerland) with the primer set LN005-LN006 (Table 1). The probe was detected using DIG Luminescent Detection Kit and CDP-star (Roche). The fragment size was inferred based on the position of the 2-Log DNA Ladder and lambda DNA-*Hin*dIII Digest (New England Biolabs) in the ethidium bromide-stained gel.

### Copy number determination of the recombination sites

**Fig 1:**
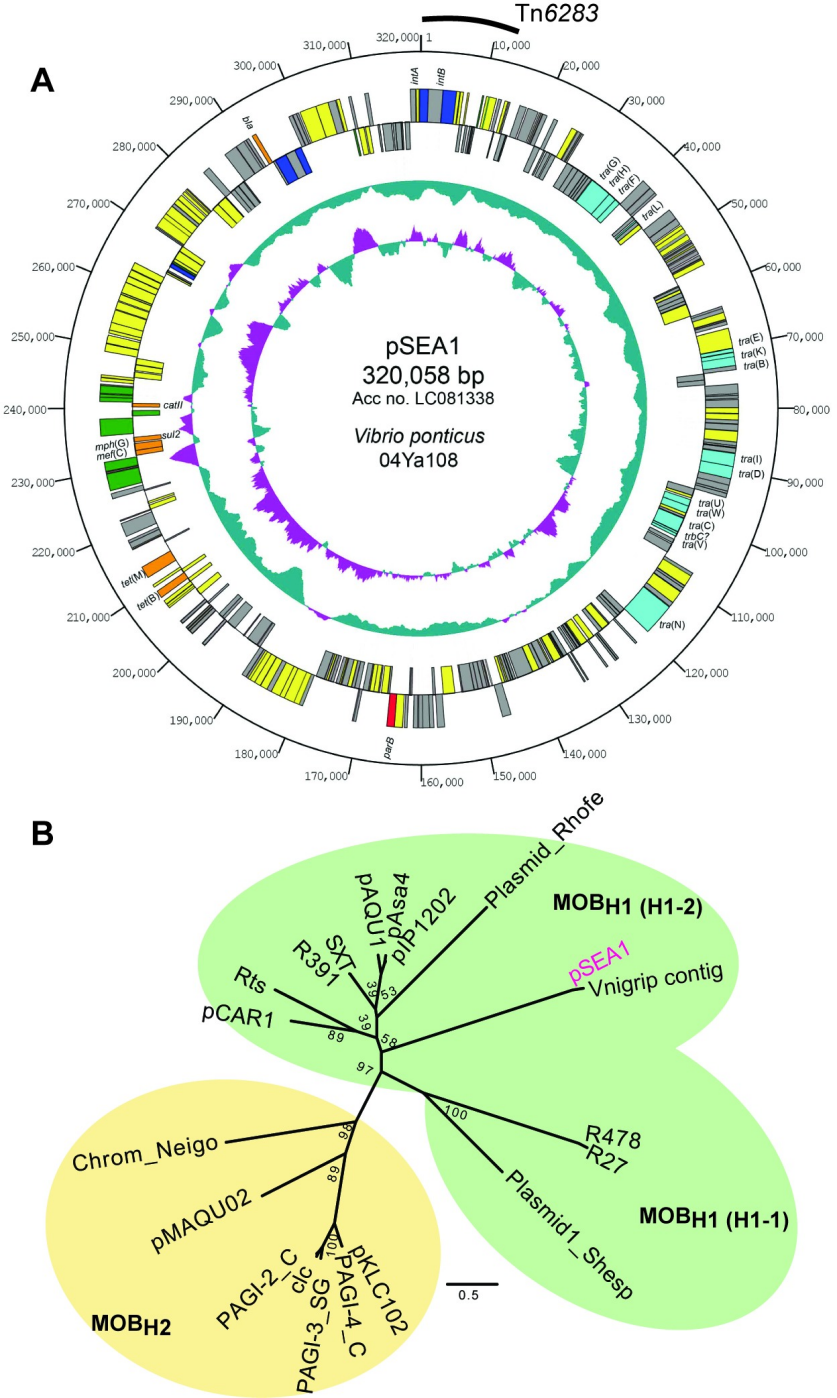
Plasmid pSEA1 from *Vibrio ponticus*. (A) Circular genetic map of pSEA1. Symbols on the circle are as follows (outside to inside): coordinates, protein-coding sequences coded clockwise, protein-coding sequences coded anticlockwise, G+C content, G+C skew. The Tn*6283* region is indicated by arc. Color codes of the coding sequences are as follows: red, partition; blue, site-specific recombination or transposition; light blue, conjugative transfer; green, transposase; orange, antibiotic resistance; yellow, other function; grey, unknown functions. (B) Unrooted phylogenetic tree of MOB_H_ family mobile genetic elements. The tree was constructed based on the maximum-likelihood method using the JTT+G model with PhyML [23].

We quantitated copy numbers of recombination sites using qPCR following absolute quantitation protocol using standard template solutions for which concentrations of target molecule included were known. Standard templates were obtained by cloning PCR products into pGEM-T easy vector (Promega Corp.). The target molecule copy number in standard solution was estimated according to absorbance at 260 nm and molecular weights. Serially diluted standard solutions were used to generate standard curves with *R*^2^ value > 0.996. The joint on the expected circular form of Tn*6283* (*att*_Tn*6283*_) in strains TJ108W0 and 04Ya108 was quantified with primers LN011 and LN012 in a 20-μl final volume on a Thermal Cycler Dice Real-Time System (Takara Bio Inc., Shiga, Japan). The program was as follows: denaturation for 5 sec at 95°C and annealing/extension for 8 sec at 64°C for the primer set 2599370F-2599572R, and 30 sec at 60°C for the other primer sets. Specific amplification was confirmed by melting curve analysis for each amplicon. Each reaction was performed with at least two technical replicates and each quantification was conducted for five biological replicates. The copy number of Tn*6283* was quantified by amplification of *intA* (Fig 1) with the primer set LN005-LN006. The total copy number of *E*. *coli* chromosome and pSEA1 was quantified by amplification of *dxs* and *traI*, with primer sets dxsF-dxsR [18] and LN009-LN010, respectively. The Tn*6283*-free integration sites resulting from Tn*6283* excision from the *E*. *coli* chromosome and pSEA1 in 04Ya108 were quantified by PCR with primer sets 2599370F-2599572R and LN015-LN016, respectively.

### Persistence of Tn*6283* in the *E*. *coli* cell population

Three replicate cultures of LN5 (*bcp*::Tn*6283*) were prepared in 5 ml LB without antibiotics, and then 5 μl of each culture was transferred into 5 ml fresh medium and incubated at 37°C for 24 hr; this cycle was repeated 10 times. Cultures at day 1, day 6, and day 11 were serially diluted in saline and propagated on LB agar to isolate colonies. The fraction of Tn*6283*-containing viable cells was monitored for 10 days (approx. 100 generations) using colony PCR for 40 colonies per culture lineage to detect “*attL*” in the chromosome.

### Growth curve analysis

Three *E*. *coli* strains, W3110, LN3 (Δ*bcp*), and LN5 (*bcp*::Tn*6283*), were cultured to the stationary growth phase, and the OD_600nm_ of the cultures were normalized to 0.3 (in 96-well microtiter plates) across samples by diluting the cultures with fresh media. The OD_600nm_-normalized cultures were again diluted 1000-fold in fresh medium, and then incubated in a 150 μl scale in a 96-well microtiter plate covered with Breathe-Easy film (Sigma-Aldrich Co. LLC., St. Louis, MO, USA) and read on a microplate reader (Sunrise^TM^ Rainbow Thermo RC-R, TECAN, Zürich, Switzerland). Incubation was continued for up to 20 hr with agitation at 37°C. The OD_600nm_ value of each well was recorded every 10 min. Growth parameters (lag phase time, maximum growth rate, maximum OD) were estimated using the smooth.spline function with the 100-times bootstrapping run option implemented in the Grofit program [20].

### Microscopy and flow cytometry

For cell morphology analysis, cells were cultured in the microplate up to an appropriate growth phase as described above. The cells were then transferred to 1.5-ml tubes, washed once with saline, and then stained with a solution containing 1 μM SYTOX-Green (Thermo Fisher Scientific, Waltham, MA USA) and 1 μg/ml DAPI (Dojindo Laboratories, Kumamoto, Japan) for 10 min. Stained cells were spotted on an agarose film prepared on slide glasses. Images were obtained using a Zeiss LSM 780 inverted microscope (Carl Zeiss, Oberkochen, Germany). The number of dead cells was estimated using 0.03 μM SYTOX-Green staining, washed, re-suspended in saline, and the number of stained cells in a total of 100,000 cells was counted using a flow cytometer (LE-SH800ZFP cell sorter, Sony Corporation, Tokyo, Japan). A 488-nm laser was used for excitation of SYTOX-Green, and the emitted signals were detected with a 525/50-nm bandpass filter. Before fluorescence analysis, the cells were gated using FSC-A and SSC-A, and possible doublet cells and cell aggregates were excluded with FSC-A and FSC-H gating (S1 Fig). Finally, data from 55,167 to 69,949 single cells at the exponential phase (4-hr incubation in the microtiter plate) were obtained per sample to estimate the proportion of dead cells in the culture. The data were analyzed using FlowJo software v10.2 (FLOWJO, LLC. Ashland, OR, USA).

### Phylogenetic analysis

Amino acid sequences of TraI homologs were retrieved from the NCBI database, considering representative homologs previously reported [14]. The TraI alignment was generated using PROMALS3D [21], and then trimmed to the N-terminal region equivalent to position 1–475 in pSEA1 TraI. The relaxase sequences from the MOB_H3_ group poorly aligned with the sequences of homologs from other MOB_H_ subgroups, and were thus omitted from the alignment to improve the accuracy of phylogenetic inference. The phylogenetic relationships among members in the MOB_H_ group were inferred based on the filtered TraI alignment (398-aa sequence without gaps) using the maximum-likelihood method and the JTT+G model in PhyML [22]. Bootstrapping was performed 100 times.

### Statistical analysis

Quantitative data were subjected to Bartlett’s test to determine the statistical significance of variance homogeneity. Comparison of means between two groups was conducted using Student’s t-test or the Wilcoxon rank sum test as appropriate. Multiple comparisons were conducted using ANOVA, Tukey test, Kruskal-Wallis test, or Steel-Dwass test. All tests were performed using the functions implemented in R version 3.3.1 (The R Foundation for Statistical Computing Platform).

### Accession number

The entire sequence of pSEA1 was deposited in GenBank/DDBJ/EMBL databases under the accession number LC081338.

## Results

### Identification of the MOB_H_ group plasmid pSEA1

To identify active MGEs in a *V*. *ponticus* aquaculture isolate, we used our previously generated transconjugant, named strain TJ108W0, from mating of *V*. *ponticus* 04Ya108 with *E*. *coli* W3110 [4]. The assembled scaffold from short reads of the TJ108W0 genome revealed a plasmid-like sequence containing 13.8-kb repeats at both ends, which did not match any *E*. *coli*-derived sequence. The terminal sequences of the reads that mapped to the edge of the scaffold were identical to the *bcp* gene sequence in the W3110 chromosome, indicating that an MGE was integrated into the *E*. *coli* chromosome. This integration was confirmed through PCR amplification and sequencing of the expected borders between the insert (MGE) and the *E*. *coli* chromosome, which revealed an approximately 346-kb insert in the chromosome. Two copies of the 13.8-kb repeat were identified at one chromosome–MGE border, and one copy of the repeat was identified at the other border (see the following sections). Southern hybridization identified a sequence equivalent to the chromosome insert existing as a plasmid in a donor *V*. *ponticus* strain (S2 Fig). We designated this chromosome insert with a single copy of the 13.8-kb region as plasmid pSEA1.

The plasmid pSEA1 was 320,058 bp in size and contained 324 coding sequences (CDSs) (Fig 1A), which were nearly symmetrically distributed; i.e., 169 CDSs were oriented clockwise and 155 CDSs were oriented counterclockwise. Two hundred two CDSs (74.9%) showed similarity to the homologs from *Vibrionaceae*, and 178 CDSs (55.1%) showed relatively high similarity to the homologs from *Vibrio nigripulchritudo*, which is a new emerging shrimp pathogen (NC_010733) [23] (S1 Dataset). Fifteen CDSs showed similarity to the type IV secretion system components, and seven CDSs showed similarity to known ARGs, including *tet*(B), *tet*(M) [24], *mef*(C), *mph*(G) [25], *sul*2 [26], *catII* [27], and a class A beta-lactamase gene [28], respectively. Except for *catII*, these ARGs were common to pAQU1 and pAQU1-like plasmids found in isolates at the same aquaculture site in Japan [4, 11], and *mef*(C) and *mph*(G) have been identified in erythromycin-resistant isolates from an aquaculture site in Taiwan and a pig farm wastewater in Thailand [29]. This array of ARGs did not exhibit the characteristic features of an integron [30].

The phylogenetic relationship of this replicon with other MGEs was inferred using the amino acid (aa) sequence of relaxase (TraI) following sequence alignment of pSEA1 TraI with homologs from the MOB_H_ family of conjugative transferable elements [14]. Conserved aa sequence motifs in the MOB_H1_ and MOB_H2_ groups were identified in pSEA1 (S3 Fig). Phylogenetic analysis based on a 475-aa alignment indicated that pSEA1 forms a subclade with a plasmid-like contig found in the draft genome of *Vibrio nigripulchritudo* strain FTn2 (Fig 1B) [31]. pSEA1 is thus a novel member of the MOB_H_ family of conjugative elements [14], and can be grouped in the MOB_H1_ subgroup with plasmids pCAR1, Rts, and SXT-related elements (Fig 1B).

### Integrative element Tn*6283*

**Fig 2:**
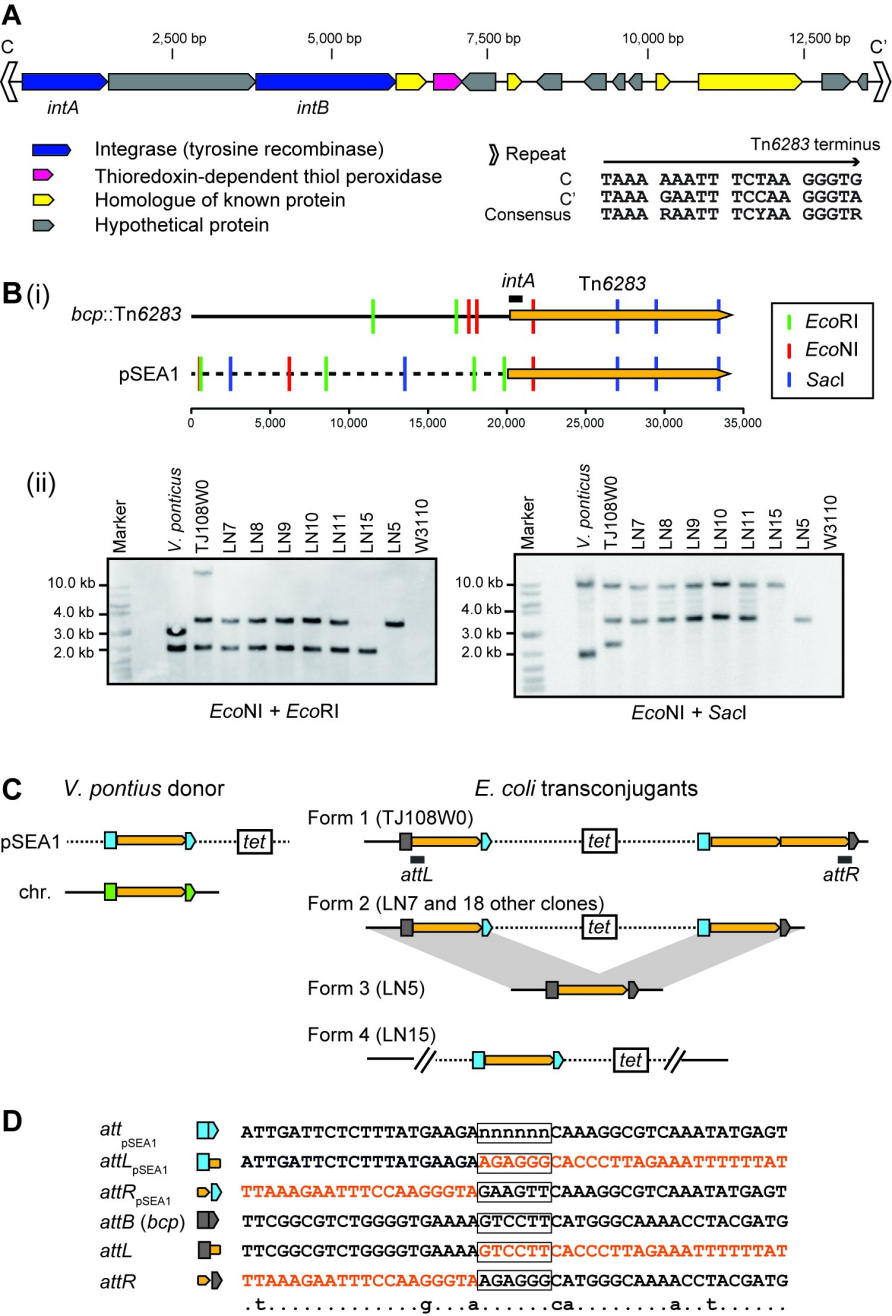
Integrative element Tn*6283*. (A) Genetic map of Tn*6283*. The color code for coding sequences is the same as shown in Fig 1. Thioredoxin-dependent thiol peroxidase, a homolog of the product of *bcp*, is shown in pink. (B) Variation of the pSEA1 integration pattern among transconjugant chromosomes. (i) Restriction map of the boundary between *bcp* and the left end of Tn*6283*, and the boundary between the pSEA1 backbone and the left end of Tn*6283*. The *E*. *coli* chromosome is shown as a solid line. The pSEA1 backbone is shown as a dashed line. The scale bar indicates the base pair position. The position at 20 kb upstream of the Tn*6283* left-end was set as position 1. (ii) Southern blots for digested genomic DNA. The *intA* region (indicated by horizontal line) was used as a probe. (C) Integration pattern of Tn*6283*. The yellow pentagon indicates Tn*6283*. Colored symbols are the split targets of Tn*6283* in pSEA1 (light blue), *E*. *coli* chromosome (gray), or *V*. *ponticus* chromosome (green). (D) Nucleotide sequences around the Tn*6283* insertion sites. The *att*_Tn*6283*_ spacer region is boxed. The nucleotide sequence moving with Tn*6283* is shown in orange. The nucleotide sequence of the central part of *att*_pSEA1_ on the ancestral pSEA1 is not known, and is therefore labeled as "n".

The 15 CDSs of the 13.8-kb repeat region identified in the transconjugant chromosome include two tyrosine recombinase genes, *intA* and *intB* (Fig 2A). The *intA* product (467 aa) showed a lambda-Int family recombinase-like secondary structure consisting of an arm-type site-binding domain at the N-terminus [32], whereas the *intB* product (731 aa) is a larger protein containing a tyrosine recombinase C-terminal catalytic domain fold (cd00397). None of the CDSs encodes homologs of known relaxases, replication proteins, or recombination directionality factor protein Xis. No apparent ARGs were detected in this region, although a Group II intron reverse transcriptase gene (locus_tag = pSEA1_013) and thioredoxin-dependent thiol peroxidase gene (locus_tag = pSEA1_005) were identified. Two imperfect 19-bp repeats were detected near the termini of this 13.8-kb region, motif C on the left end (Fig 2A) and motif C’ on the right end, which are expected to function as core sites for site-specific recombination [33]. Since the 13.8-kb region could be excised from the plasmid pSEA1 and inserted into the *E*. *coli* chromosome, it was designated as a novel integrative element, Tn*6283*, with approval of the Tn number at the transposon registry site (http://transposon.lstmed.ac.uk/). Since the *intA* product was predicted to possess an extended N-terminus as Lambda Int, the Tn*6283* region may contain motifs conserved in representative ICEs and phages; e.g., an integrase-binding arm-type site, integration host factor (IHF)-binding sites, and Xis-binding regions [33, 34]. However, these motifs could not be identified in the Tn*6283* region based on the nucleotide sequence.

### Tn*6283* integrates into a specific site in *E*. *coli*

An integration site of pSEA1 with an additional copy of Tn*6283* in the chromosome was initially revealed by genome sequencing, and a target site was identified in the *bcp* gene that encodes thiol peroxidase (Fig 2B). We next investigated (i) whether the integration of pSEA1 into the *E*. *coli* chromosome is reproducible, and (ii) whether the *bcp* gene is a preferred target site for Tn*6283*.

One tetracycline-resistant transconjugant was selected from each of 20 independent mating experiments between *V*. *ponticus* 04Ya108 and *E*. *coli* W3110, and the integration of pSEA1 into the chromosome was by PFGE and subsequent Southern hybridization (S2 Fig). Thus, pSEA1 integration into the recipient chromosome was reproducible. However, no transconjugants showing the tetracycline-resistance phenotype were obtained when the *E*. *coli* transconjugant strain TJ108W0 was used as a donor and *E*. *coli* strain W3110Rif^r^ was the recipient. This suggested that pSEA1 cannot replicate in *E*. *coli*, and that its transfer function is not active once integrated in *E*. *coli*.

We next investigated variation of the integration pattern of pSEA1 and Tn*6283* in the transconjugant chromosome. We chose seven transconjugants (TJ108W0, LN7, LN8, LN9, LN10, LN11, LN15) for this analysis, and conducted Southern hybridization for their digested genomic DNA using the *intA* region as a probe (Fig 2B). The results suggested the existence of three pSEA1 integration forms (Fig 2C). The first form, form 1, was detected in TJ108W0, which was subjected to genome sequencing. Three Tn*6283* copies were present in TJ108W0 as expected from the sequence data (Fig 2B). The second form, form 2, was detected in five clones (LN7, LN8, LN9, LN10, LN11), which carried two Tn*6283* copies. In LN15, only one *intA* copy was present (form 4 in Fig 2B). Subsequent PCR analysis revealed that the intact *bcp* gene and two pSEA1 backbone–Tn*6283* borders (*attL*_pSEA1_, *attR*_pSEA1_) were present in LN15 (B in S4 Fig, pattern C). Therefore, Tn*6283* did not move from the original locus and is therefore unlikely to be involved in pSEA1 integration of the chromosome upon the generation of LN15.

The integration forms of pSEA1 in the remaining 14 transconjugants were analyzed by long PCR, assuming form 1, form 2, or form 4 (Fig 2B and S4 Fig). Since homologous recombination can occur between Tn*6283* copies in form 1 and form 2, cells carrying a single copy of Tn*6283* (form 3 in Fig 2C) can emerge in the cell population. PCR detection of the Tn*6283*–chromosome borders suggested that the cell populations of the remaining 15 transconjugants contained a mixture of form 2 and form 3 (B in S4 Fig, pattern A), whereas the cell population of TJ108W0 contained a mixture of all expected forms (S4 Fig). The tandem repeat structure of Tn*6283* in form 1 can be explained by additional excision and insertion of Tn*6283* into *attR* in form 2. Collectively, these results indicate that Tn*6283* mainly targets the *bcp* gene when *E*. *coli* is used as recipient. The integration site in the *bcp* gene did not contain sequence motifs such as an inverted repeat and direct repeat, and showed no homology to repeat sequences (C, C’) in Tn*6283*; however, the central part of the target sequence tended to be flanked by A at the 5′ end and by CA at the 3′ end (Fig 2D).

These analyses also revealed that *V*. *poniticus* strain 04Ya108 carries two copies of Tn*6283*, one in pSEA1 and the other in the chromosome (Fig 2B). However, the precise insertion position of Tn*6283* in the *V*. *ponticus* chromosome is currently unclear.

### Resistance gene integration requires a homologous recombination system

**Fig 3:**
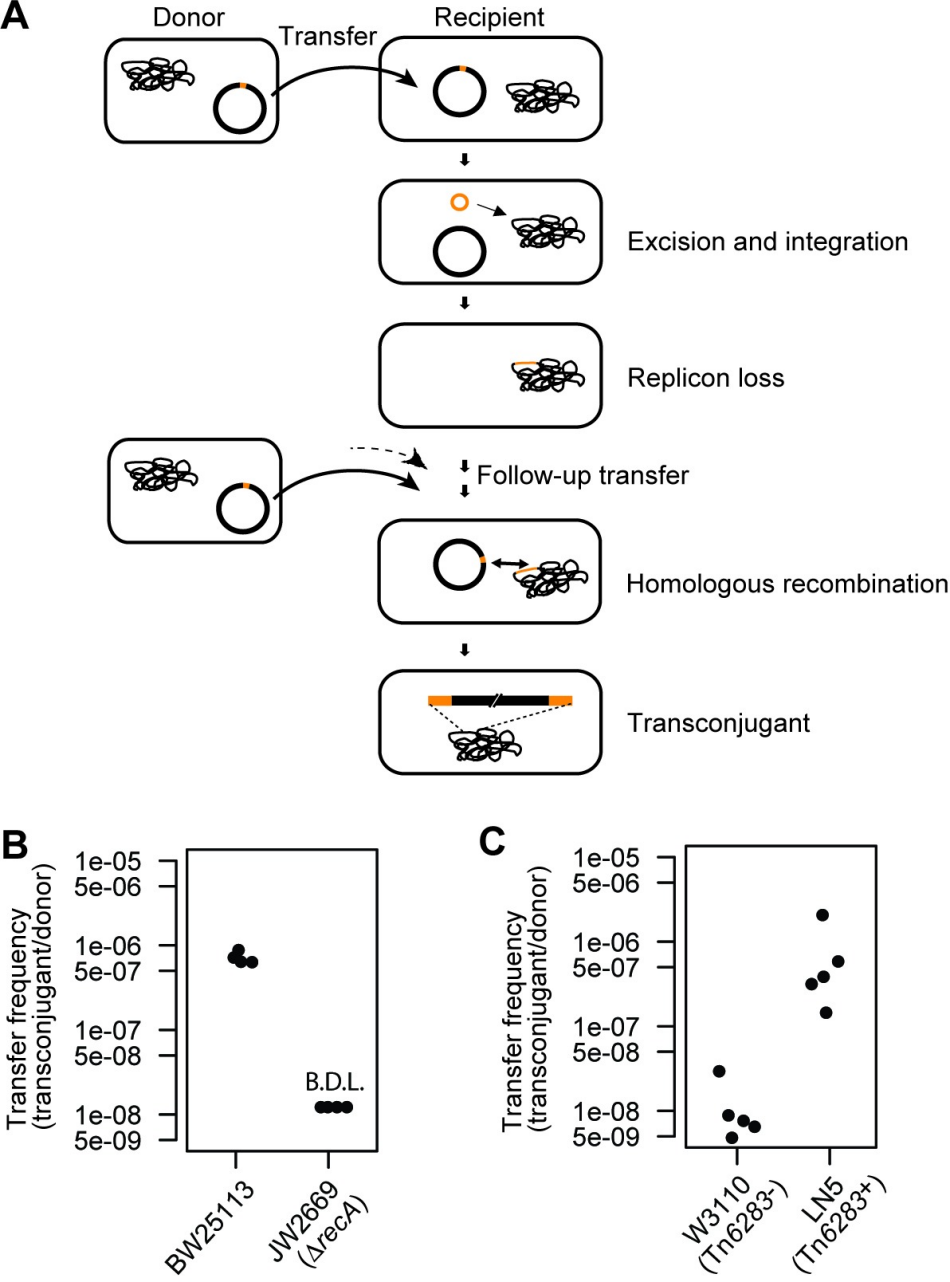
Interplay of a non-conjugative integrative element and a conjugative plasmid in interspecies antibiotic resistance gene transfer. (A) Two-step gene transfer model. First, the non-conjugative integrative element is transferred to a recipient cell via suicidal plasmid transfer and is then excised and integrated into the recipient chromosome. The subsequent plasmid transfer allows for integration of the plasmid backbone carrying antibiotic resistance genes into the recipient chromosome via homologous recombination using the homology of the integrative element. The diagram follows the canonical ICE excision model. (B) RecA-dependence of ARG transfer from *V*. *ponticus* to *E*. *coli*. The data were obtained from four independent mating assays. (C) Tn*6283*-dependence of ARG transfer from *V*. *ponticus* to *E*. *coli*. The data were obtained from five independent mating assays.

Since the most common pSEA1 integration form, form 2 (Fig 2C), can be formed through homologous recombination between one copy of Tn*6283* integrated into the chromosome and the other copy on the circularized pSEA1, the observed interspecies ARGs transfer was hypothesized to involve two types of recombination with support of the DNA mobilization function of the conjugative plasmid: (i) suicidal transfer of pSEA1 followed by excision and integration of Tn*6283*, and (ii) follow-up suicidal transfer of pSEA1 and its chromosomal integration using the homologous recombination system of the recipient cell (Fig 3A). A structure similar to from 2 can be formed by replicative transposition such as that mediated by Tn*3* transposase [35]. However, Tn*3*-like replicative transposition is unlikely in this case because Tn*6283* transposition seems to occur through its circle formation (see below). To test whether the homologous recombination system was actually involved in the integration of pSEA1 into the chromosome and the resulting interspecies transfer of ARGs, we used the *recA*-null mutant (JW2669) as a recipient strain, and then compared the transfer frequency of the *tet*(M) gene between the wild-type and mutant recipient. Besides its *recA*-null status, JW2669 is otherwise isogenic with BW25113. When the parental strain BW25113 was used as a recipient, the resistance gene transfer was detected at a frequency of 7.2 × 10^−7^ copies per donor (Fig 3B). However, the transfer frequency was under the detection limit (<1.2 × 10^−8^ copies per donor) when strain JW2669 was used as the recipient.

We next tested whether the presence of a Tn*6283* copy in the recipient chromosome can increase the frequency of pSEA1 integration using *E*. *coli* strain LN5 and its isogeneic parent strain W3110 as recipient. Strain LN5 carries a single copy of Tn*6283* in the *bcp* gene, which was obtained by serial batch-culture transfer of one Tc^r^ transconjugant in a non-selective medium. Although the transfer of the resistance gene was detected for both strain W3110 (Tn*6283*^-^) and LN5 (Tn*6283*^+^), the transfer frequency was about 60-fold higher in LN5 than that in W3110 (N = 5, P = 0.007937 in a two-sided Wilcoxon rank sum test; Fig 3C).

Collectively, these results suggest that the integrative transfer of pSEA1 from *V*. *ponticus* into the *E*. *coli* chromosome highly depends on the homology of the Tn*6283* copy, as well as on the homologous recombination system of the recipient cell.

### Tn*6283* circle originates from one specific strand

**Fig 4:**
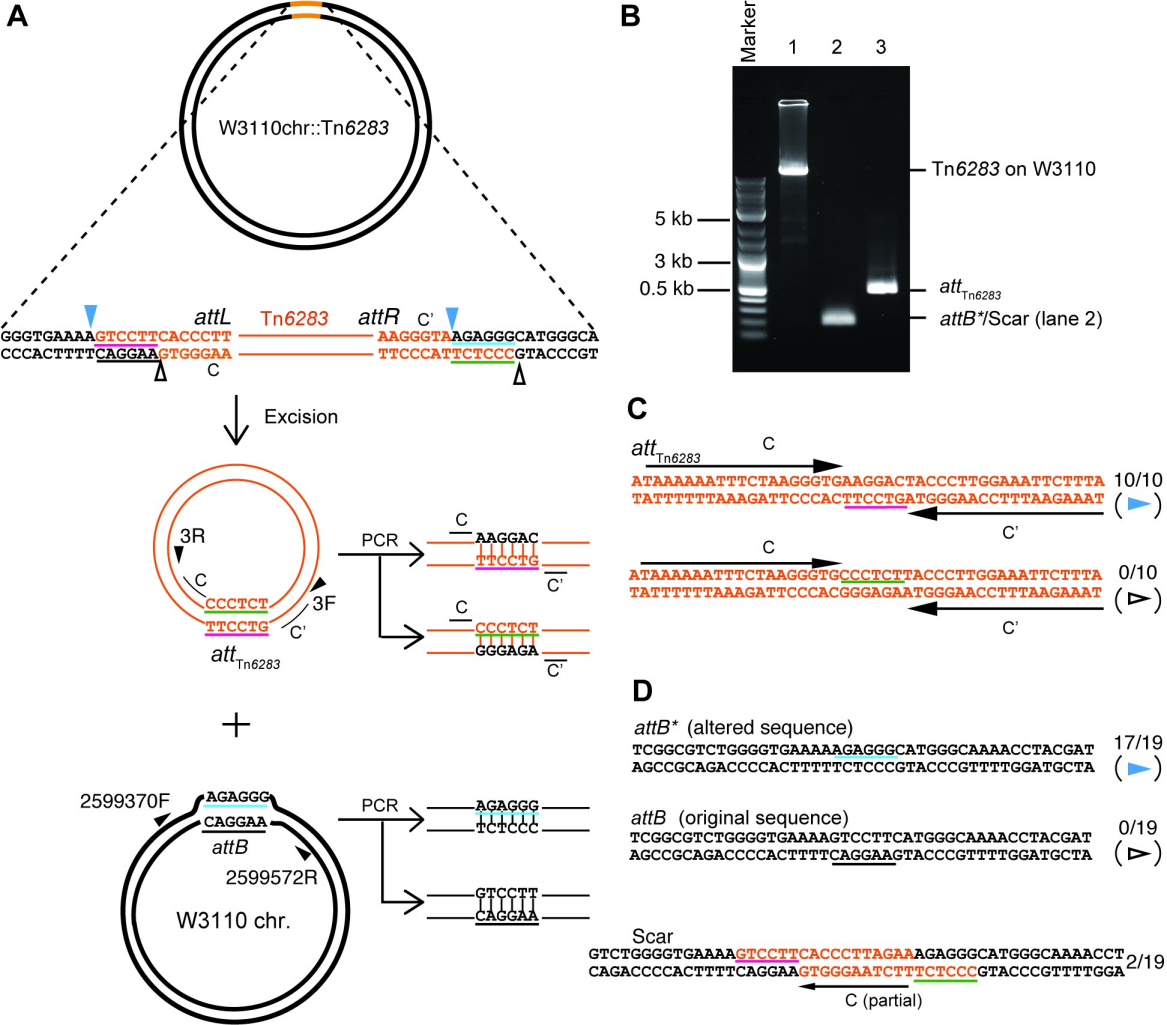
Excision of Tn*6283* from the *E*. *coli* chromosome. (A) Design for PCR detection of recombination products. The diagrams show the hypothetical scenario in which Tn*6283* excises itself as a circular molecule and forms a heteroduplex joint, while the Tn*6283* donor site also forms a heteroduplex joint. The PCR-amplified heteroduplex joints should contain two types of sequences in the spacer between terminal repeat sequences. (B) PCR detection of joint formation on the recombination products. Lane 1: long PCR designed to detect the occupied Tn*6283* donor site (primer set 2599572R-2599370F). Lane 2: detection of unoccupied Tn*6283* donor sites (primer set 2599572R-2599370F). Lane 3: detection of circularized Tn*6283* (primer set 3F-3R). (C) Sequences of PCR-amplified joints on the circularized Tn*6283*. The observed frequency is indicated next to each sequence. (D) Sequences of PCR-amplified joints on the unoccupied Tn*6283* donor sites. In the upper panel, two types of expected joint sequences are shown. The lower panel shows the unexpectedly observed sequence, designated Scar.

Since Tn*6283* contains two tyrosine recombinase genes, it would be expected to excise itself in the typical ICE circular form [34]. To precisely define the mobility unit of Tn*6283*, we determined the sequence of the expected recombination joint “*att*_Tn*6283*_” formed upon excision of the element. In addition, we investigated how the Tn*6283* insertion site in the donor replicon pSEA1 is joined upon excision of Tn*6283* by determining the sequence of the hypothetical recombination joint "*att*_pSEA1_"_._ For this analysis, we used *E*. *coli* strain LN5 that carries a single copy of Tn*6283* (Fig 4), and *V*. *ponticus* harboring pSEA1 (S5 Fig).

In the *E*. *coli* chromosome, motifs C and C’ of Tn*6283* were not flanked by a direct repeat (Fig 2D). Thus, *att*_Tn*6283*_ was initially hypothesized to contain a heteroduplex spacer (Fig 4A), equivalent to the “coupling sequence” of Tn*916* [36, 37]. Excision of Tn*6283* from the *E*. *coli* chromosome integration site (*attB* within the *bcp* gene) was detected (Fig 4B). The PCR products were cloned into the pGEM-T easy vector and sequenced to determine the spacer sequence. The *att*_Tn*6283*_ on the excised form of Tn*6283* consisted of motif C, a 6-bp spacer (AAGGAC), and C’ (Fig 4C). Thus, the termini of Tn*6283* in the *E*. *coli* chromosome were 6 bp downstream of the 3’ end from C and C’. These results indicated that Tn*6283* is excised as a circle by introducing a nick at a position 6 bp from the two terminal repeats. Although two types of *att*_pSEA1_ sequences were expected, the ten cloned PCR products contained only one type (Fig 4C), which could be generated by strand transfer between two nicked sites on the top strand in Fig 4A (blue arrowheads). This suggests that the excised Tn*6283* circle carries a homoduplex (complementary sequences) at the spacer region, and thus strand transfer preferentially occurs on one specific strand of the donor molecule.

The original *attB* sequence, before the Tn*6283* insertion, can be generated by recombination between “*attL”* and “*attR”* (Fig 4A). The PCR product with the expected size of *attB* was detected at a relatively low level (Fig 4B). Seventeen of the 19 clones from PCR products expected to carry the *attB* sequence contained an *attB* variant, designated attB*, that contained a 6-bp spacer region originating from one terminus of Tn*6283* on pSEA1. This product was also generated by strand transfer between two nicked sites in the top strand (Fig 4A and 4D blue arrowheads). Two clones showed the scar sequence (Scar in Fig 4D), containing of a partial (11-bp) sequence of motif C. This scar sequence is not the product generated by strand exchange between the two primary nicking sites (Fig 4A). In other word, the detected joints on the two products (Fig 4C and 4D bottom) were not the products derived from one excision event.

Tn*6283* is inserted into a hypothetical protein gene on pSEA1 in *V*. *ponticus*, whose homolog was found in *V*. *nigripulchritudo*, and motifs C and C’ of Tn*6283* were not flanked by a direct repeat (A, B in S5 Fig). Sequencing of the PCR products of the expected recombination joint *att*_Tn*6283*_ in five clones showed that the *att*_Tn*6283*_ sequence contained motifs C and C’ and two 6-bp sequences (CCCTCT, AACTTC) between the two motifs, which were identical to the 6-bp sequences next to C and C’ on pSEA1 (A, C, D in S5 Fig). We could not determine whether the circularized Tn*6283* originated from its copy in the chromosome, pSEA1, or both (Fig 2D). The empty donor site, *att*_pSEA1_, was not as efficiently amplified as *att*_Tn*6283*_ in PCR (B in S5 Fig). The cloned fragment of *att*_pSEA1_ contained scar sequences that include entire motif C (F in S5 Fig), similar to the case for *attB* in *E*. *coli* (Fig 4E). These results confirm that Tn*6283* circularizes itself upon its excision but does not mediate equivalent strand exchange for the replicon side on the substrate *in vivo*.

### Tn*6283* excision does not generate an unoccupied donor site *in vivo*

**Fig 5:**
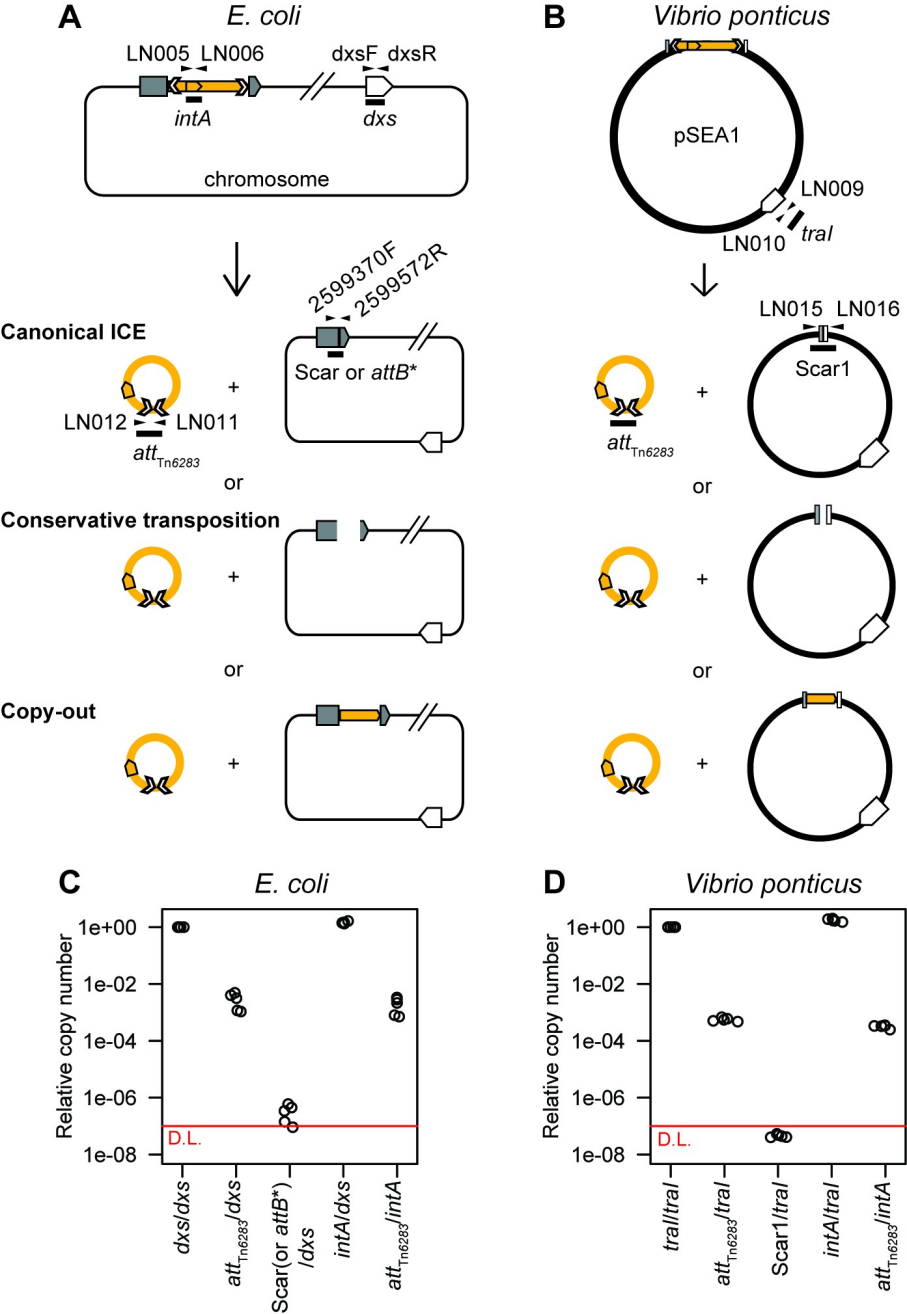
Tn*6283* excision barely generates unoccupied donor sites *in vivo*. (A) A model for Tn*6283* excision in *E*. *coli* strain LN5. Three models are proposed (see main text for details). Primer sets used for qPCR are indicated as arrows. (B). A model for Tn*6283* excision in *V*. *ponticus*. (C) Copy numbers of recombination joints in *E*. *coli*. Copy numbers of two recombination joints (*att*_Tn*6283*_, attB*) shown as the ratio of the number of joints to the number of replicon backbone (*dxs*) or total Tn*6283* molecule (*intA*). In one sample, the copy number of attB* was below the detection limit. (D) Copy numbers of recombination joints in *V*. *ponticus*. Copy numbers of two recombination joints (*att*_Tn*6283*_, Scar1) shown as the ratio of the number of joints to the number of pSEA1 backbone (*traI*) or total Tn*6283* molecules (*intA*). Each dot indicates total DNA extracted from one batch of stationary-phase cultures. The amount of Scar1 was too low to detect in a quantitative manner (outside the range of standard curve), and is thus shown as the numbers below the detection limit (red line: 10^−7^).

Since the unoccupied Tn*6283* donor sites (attB*, *att*_pSEA1_) were detected at low levels in the Tn*6283*-containing cell population, and scar sequences were detected as the recombination joints on the donor site, we hypothesized that the replicon backbone is not efficiently joined upon Tn*6283* excision (Fig 5A, conservative transposition) or that Tn*6283* is excised via a copy-out mechanism, which leaves the original copy upon excision (Fig 5A, copy-out model). To test this idea, we quantified the copy numbers of, *intA* (total Tn*6283* copies), *att*_Tn*6283*_ (joint on the circularized Tn*6283*), *dxs* (*E*. *coli* chromosome), attB* (joint formed on the chromosome side of the recombination products in the *E*.*coli* strain), *traI* (a reporter for total pSEA1 copies in the *Vibrio* strain), and Scar1 (joint formed on the pSEA1 side of the recombination products in the *Vibrio* strain) using qPCR.

In *E*. *coli*, the mean copy number of *att*_Tn*6283*_ was 2.9 × 10^−3^ per total chromosome copies and 2.0 × 10^−3^ per total Tn*6283* copies (Fig 5D). The mean copy number of the joints on the replicon (attB*) was 3.8 × 10^−7^ per total replicon copies (Fig 5C). Thus, the copy number of the unoccupied donor site was below 0.1% of the joints on the excised Tn*6283* molecule. In *V*. *ponticus* stationary cultures, the mean copy number of *att*_Tn*6283*_ on the circularized Tn*6283* was 5.6 × 10^−4^ per total pSEA1 copies and 3.2 × 10^−4^ per total Tn*6283* copies (Fig 5D). Scar1 was detected at a low level, and the product-to-substrate ratio (Scar1/*traI*) was below the detection limit of quantitative analysis (<1 × 10^−7^ per replicon copies) (Fig 5D). Again, the copy number of unoccupied donor sites was below 0.1% that of the joint on the excised Tn*6283* molecule *in vivo*. These results suggest that the majority of the products of the replicon side were not joined, and were eventually lost from the cell population, or that Tn*6283* is excised via a mechanism similar to the copy-out-paste-in transposition model of an insertion sequence (Fig 5B).

To test whether Tn*6283* excision is growth phase-dependent, we conducted the same qPCR experiment using *E*. *coli* cell cultures at three different growth phases (S6 Fig). The results indicated that the product-to-substrate ratios *in vivo* were consistent across growth phases, and the excision frequency deduced from the *att*_Tn*6283*_/*traI* ratio was 4.1 × 10^−3^ to 5.1 × 10^−3^ (S6 Fig).

### Tn*6283* has a marginal negative fitness effect

**Fig 6:**
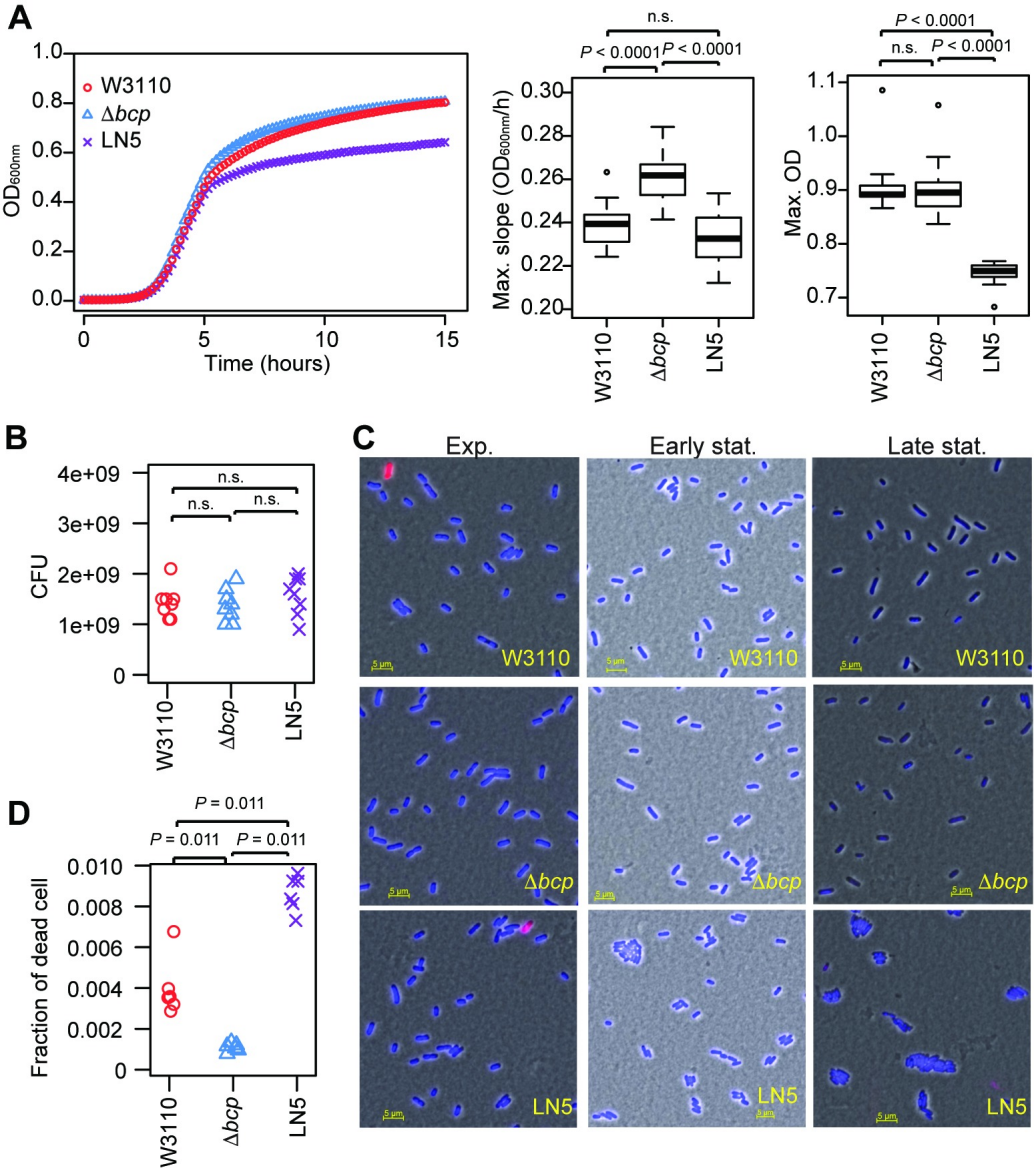
Tn*6283* does not impose a detectable fitness cost on the recipient host. (A) Growth curve of three *E*. *coli* strains cultured in LB. Left: culture OD over 15 hr. The data points represent the means from 16 growth curves. Center: box plot showing the maximum growth rate. Right: box plot showing the maximum OD values. Data were compared among three groups using ANOVA and Tukey’s post-hoc test. (B) Colony-forming units of stationary-phase cultures. Data were obtained from cultures grown in eight distinct wells in 96-well microtiter plates. (C) Cell morphology at three different growth phases. Left: exponential phase (equivalent to 4 hr in the growth curves shown in (A)). Middle: early stationary phase (approximately 7 hr). Right: late stationary phase (20 hr incubation in a microtiter plate). Cells were stained with DAPI (blue) and SYTOX-Green (red). (D) Fraction of dead cells in the exponential phase. Cells in the exponential phase were stained with SYTOX-Green without fixation, and then fluorescence was detected using flow cytometry. The data were filtered to select only single cells using FlowJo software. The data were compared among three groups using the Steels-Dwass test.

Since the transfer of mobile DNA into new hosts can have negative impacts on fitness [38, 39], we next evaluated whether the integration of Tn*6383* into *E*. *coli* imposes a detectable fitness cost (assessed by growth parameters) on the recipient strain, which would potentially arise from its constant excision and the expression of genes embedded in the integrative element. There was no significant difference in the maximum growth rate between the parental strain W3110 and strain LN5 carrying Tn*6283* (Fig 6A), indicating that Tn*6283* does not negatively influence growth of the host. However, the *bcp*-knockout strain LN3 (Δ*bcp* strain) showed a higher growth rate compared to that of the other two strains (*P* < 0.0001 for both comparisons in a Tukey test). The maximum culture optical density (OD) was significantly lower in LN5 compared with that of the other two strains (Fig 6A) (*P* < 0.0001 for both comparisons in the Tukey test). There was no detectable difference in the number of colony-forming units (CFUs) among the three strains (Fig 6B; *P* = 0.45 in ANOVA), indicating that the relatively low OD of LN5 was not due to cell death. However, LN5 contained a considerably higher number of cell aggregates compared with the other strains at the late stationary phase according to microscopy observation (Fig 6C), suggesting that its decreased OD was likely due to cell aggregation, which was either a direct or indirect effect of having Tn*6283* inserted at the *bcp* locus in this strain.

The excision frequency of Tn*6283* was estimated to be 5.0 x 10^−3^ ± 0.1 x 10^−3^ (n = 5) per replicon at the exponential phase in the LN5 cell population (Fig 5B). If the excision of Tn*6283* accompanies double-stranded breaks of the Tn*6283*-carrying chromosome, the Tn*6283*-containing cell population would contain relatively more dead cells than the other populations. Indeed, flow cytometry observation of dead-stained cells was consistent with this hypothesis. The fraction of dead cells was lowest in Δ*bcp* (0.0011 ± 0.0002) (Fig 6D), whereas the highest fraction was detected in LN5 harboring Tn*6283* (0.0087 ± 0.0009); the fraction of dead cells of W3110 was intermediate between the other two strains (0.0040 ± 0.0014).

This low burden could explain the stable maintenance of Tn*6283* in the cell population despite its constant generation of the empty donor site at a low frequency. Thus, we tested whether Tn*6283* can be stably maintained in a host cell population using *E*. *coli* LN5 as the model host. In three replicate assays, we did not detect the emergence of Tn*6283*-free colonies (S7 Fig). This confirms that Tn*6283* can persist in the cell population without particular selection for its carriage once it has integrated into the genome.

## Discussion

Despite increasing concern on the accumulation of ARGs in aquaculture, little is known about the genetic entities and mechanisms involved in gene transfer in these environments [40]. The current paradigm is that the dissemination of ARGs is largely mediated by MGEs that support intercellular mobility, such as autonomously replicating conjugative plasmids and ICEs represented by SXT/R391-elements, CTn*DOT*, and Tn*916* [3, 34, 41]. Dissemination of ARGs is also mediated by the interplay between the MGEs that encode conjugation factors and other sets of mobile elements that exhibit intracellular mobility, such as integron gene cassettes, mobilizable plasmids, and mobilizable integrative elements [30, 42, 43, 44]. However, these concepts of HGT have been established based on findings from human pathogens, and may not fully explain the pattern of emergence of antibiotic-resistant bacteria in aquaculture environments [4]. Here, we identified a new pattern of ARG transfer from an aquaculture isolate.

Previous studies have demonstrated the integration of a foreign plasmid into the recipient chromosome in actinomycetes [45], *Myxococcus xanthus* [46], *Acidiphilium facilis* [47], and *Acidocella* sp. [48]. Although some of the plasmids reported above are now known to be ICEs with replication function [49, 50], the underlying plasmid integration mechanism of the others has thus far remained elusive. Based on our results with plasmid pSEA1 that was integrated into the *E*. *coli* chromosome [4], we can suggest a two-step gene integration model, in which a conjugative plasmid integrates into the chromosome using the homology of an integrative element. The transposition of insertion sequences should also mediate integration of a plasmid, as known for the generation of Hfr strains in *E*. *coli* [51]. Such integrative elements can provide a larger homology stretch than the insertion sequences (1–3 kb in length). Our findings suggest that even an integrative element without mobilization function could assist with HGT in the presence of other MGEs.

Among the integrative elements that encode a tyrosine recombinase, some mainly target sequences that are identical or highly homologous to the spacer sequence in the *attP*-equivalent site [52, 53, 54] and generate a direct repeat when in the integrated form. Conversely, Tn*916*, CTn*DOT*, and SXT/R391 elements do not integrate into the sequences that show homology to the *attP*-equivalent site [34, 36, 55]. Their excision generates a heteroduplex spacer (also called a coupling sequence) at both *att* sites, one on the excised integrative element and the other on the donor site [36, 37]. This is somewhat similar to the detected behavior of Tn*6283*, which integrates into the *bcp* gene that also has no homology to the *att*_Tn*6283*_ spacer. However, there are at least a few notable differences in the excision mode between the well-characterized elements (SXT, Tn*916*, CTnDot) and the newly identified element Tn*6283*.

First, Tn*916* and SXT generate unoccupied donor sites at a comparable frequency to the generation of circularized elements [56, 57], whereas these sites are barely generated by Tn*6283* (i.e., 7,000 copies of *att*_Tn*6283*_/the empty donor site in *E*. *coli*). Furthermore, the detected joint on the donor site showed a high frequency of scar sequences, which would result in damage to the functionality of the target gene. The generation of scar sequences may be due to incorrect excisions occurring at a low frequency.

**Fig 7:**
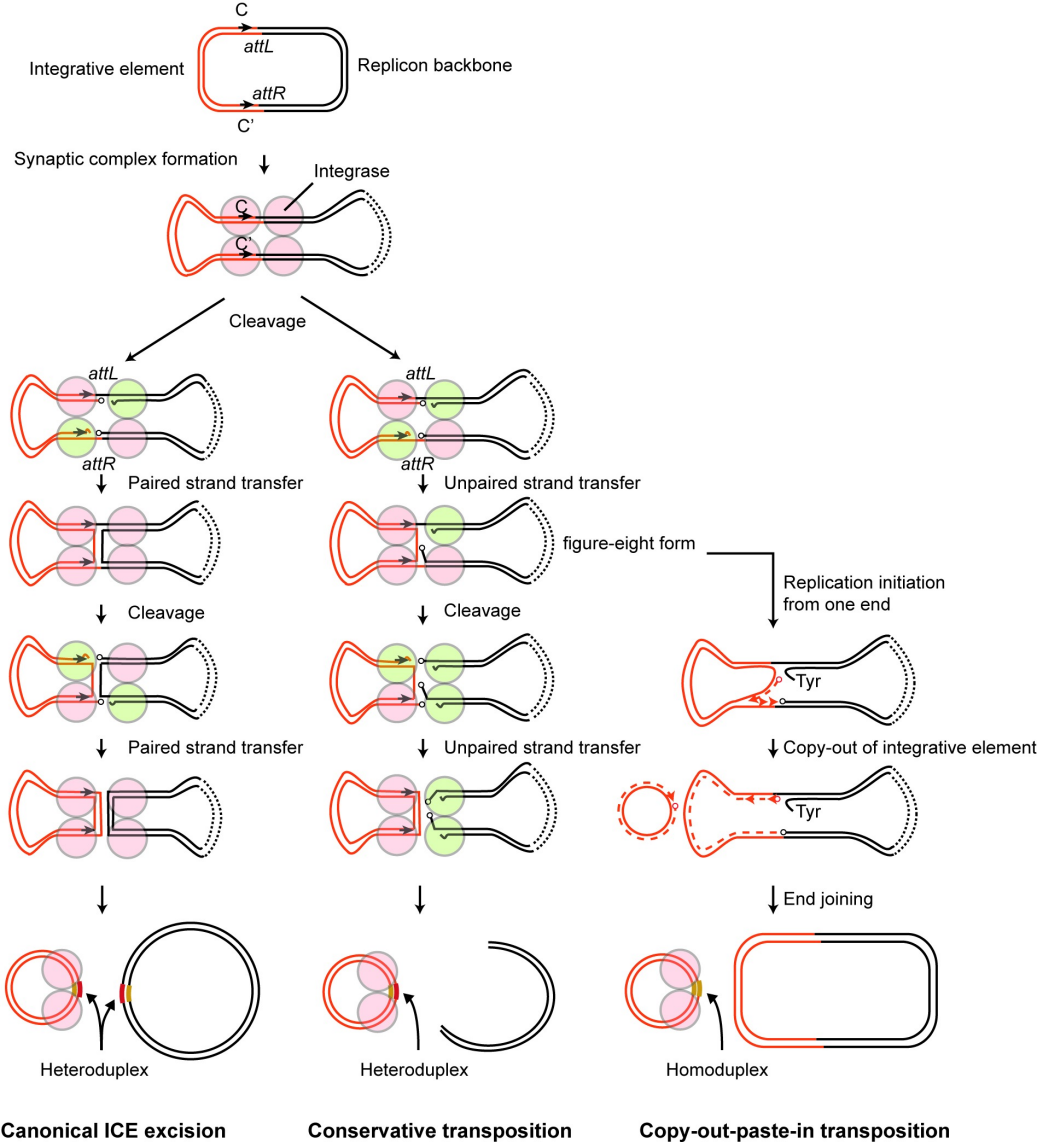
Models for Tn*6283* excision pathway. (Left) Canonical ICE excision. Red: inactive tyrosine recombinase protomer; green, active recombinase protomer. Two rounds of paired strand transfer separate the integrative element and replicon backbone. (Middle) Conservative transposition. Strand transfer occurs on only one strand upon every strand cleavage reaction. This leads to a double-strand break of the donor replicon, similar to the transposition of IS*10* and Tn*7* [60, 61]. (Right) Copy-out-paste-in transposition. Strand synthesis after the first strand transfer excises the integrative element without cleaving one strand of the parental molecule. Progression of the replication fork generates the circular form of the integrative element, leaving its original copy in the donor molecule.

Second, excised Tn*6283 in vivo* does not seem to carry a heteroduplex joint at *att*_Tn*6283*_, and instead contains a homoduplex joint according to the sequencing results of ten cloned *att*_Tn*6283*_-derived fragments (Fig 4D). Based on this evidence, we propose two excision models for Tn*6283*, which contrast with the canonical ICE excision model. One is a conservative transposition model, in which a Tn*6283* circle is generated after two rounds of unpaired strand transfer (Fig 7, middle). The other is a copy-out-paste-in transposition model, in which the replication machinery is assembled at the recombination joint and the replication fork proceeds in one direction after the first unpaired strand transfer (Fig 7, right). The latter model is employed by several IS families [58]. Since the 5′ overhangs of two Tn*6283* ends were not complementary, two rounds of strand transfer should generate a heteroduplex joint at *att*_Tn*6283*_ (Fig 7, left and middle). Although the number of sequenced clones was limited, the absence of sequence heterogeneity in the *att*_Tn*6283*_ region in strain LN5 (one copy of Tn*6283*) suggests that the Tn*6283* circle is replicated out immediately *in vivo* after the first unpaired strand transfer occurring between two sites on one specific strand of the donor molecule (Fig 4A, blue arrowheads; Fig 7, right). Together, these results suggest that copy-out-paste-in transposition is more likely than conservative transposition as an excision model for Tn*6283*.

The detailed mechanisms underlying the strand bias of Tn*6283* excision remain to be investigated. Generation of scar sequences occurring at the empty donor site suggests that nicking at *attR* (Fig 4D and 4F in S5 Fig) is a concrete process, whereas nicking at *attL* is not. The secondary nicking site was located near motif C in both *E*. *coli* and *V*. *ponticus* (A in S5 Fig, red arrows). Moreover, a motif C-like sequence, named C1, is present near motif C (A in S5 Fig), and 14 of 19 bp are conserved between motif C and motif C1. This situation is reminiscent of the “nearly precise excision” of Tn*10*, which involves internally located inverted repeat sequences [59]. We speculate that the IntA-binding to motif C1 or another recombinase IntB may be involved in generation of the scar sequence (G in S5 Fig) as well as in the strand bias of Tn*6283* excision. These hypotheses will be tested in future research.

Most ICEs such as Tn*916*, SXT, and CTn*Dot* carry a gene encoding recombination directionality factor (RDF) to control excision and integration, whereas Tn*6283* does not encode obvious RDF homologs. Furthermore, Tn*6283* does not apparently encode mobilization proteins. These features collectively suggest that Tn*6283* is a more primitive integrative element that behaves similarly to cut-and-paste or copy-and-paste type DNA transposons.

Based on the growth assay and cell morphology analysis, Tn*6283* does not seem to negatively affect fitness of recipient *E*. *coli*, at least under the laboratory condition (incubation in Luria-Bertani medium with extensive agitation), although it did show effects on cell physiology. Tn*6283* encodes a thioredoxin-dependent thiol peroxidase, which also happens to be the product of the *bcp* gene [62], although the protein identity between the two products was only 54.8% (90.3% similarity). Thus, gene inactivation caused by Tn*6283* integration may be in part compensated by the genetic cargo in Tn*6283*. This speculation is congruent with the evidence that the Δ*bcp* strain showed a deviated maximum growth rate compared with that of the wild-type strain, whereas the wild-type and LN5 strains exhibited indistinguishable growth rates. This possible gene inactivation and its compensation is analogous to the method of a family of ICEs called *recA*-mobile elements (RME) discovered in *V*. *cholera*, which disrupt the host’s *recA* gene, but complement the functional loss through a divergent *recA* homolog embedded within the elements [63]. Sequence comparison between pSEA1 and a plasmid-like contig from *V*. *nigripulchritudo* strain FTn2 revealed that Tn*6283* disrupted one hypothetical protein gene that was likely present in the pSEA1 ancestor (B in S5 Fig). However, we currently cannot determine whether such functional compensation occurred on pSEA1.

pSEA1 was classified into the MOB_H_ group of MGEs [14]. Of the six MOB families identified to date, MOB_H_ contains the smallest number of plasmids [14]. Some MGEs detected from aquatic isolates are included in the MOB_H_ group, such as A/C plasmids and the SXT/R391 family ICEs detected in marine isolates belonging to the families *Enterobacteriaceae* and *Vibrionaceae* [4, 42, 64, 65]. Further, pAQU1 and its relatives have been found in multidrug-resistant isolates from an aquaculture site [4]. pASa4 [66] and Plasmid1 [67] were discovered from the salmon pathogen *Aeromonas salmonicida* and *Shewanella* sp. isolated from an eel pond, respectively. Thus, members of the MOB_H_ group are likely one of the greatest contributors to the distribution of ARGs among *Gammaproteobacteria* in the aquatic environments. We also found *intA* and *intB* homologs in *Vibrio* and *Photobacterium* species in the NCBI database (S1 Dataset), suggesting that related integrative elements may be distributed among *Vibrionaceae* nested in the sea. A previous report indicated that approximately 10^10^ copies of the 16S rRNA gene were present in 1 g of aquaculture sediments [68]. This suggests that bacteria reside in the aquaculture sediment at a high density, comparable to the condition used in our mating assays. Thus, the interplay between primitive integrative elements and conjugative elements may contribute to the dissemination of resistance genes in the sea and to interspecies gene transfer among prokaryotes.

## Supporting information

S1 Dataset Annotation of plasmid pSEA1. (XLSX)

S1 Fig Selection of single cells in flow cytometry analysis. One representative data of W3110 (A), LN3 (B), and LN5 (C). Number of fluorescence-signal positive dead cells in the boxed area was count and summarized in (D).
(PDF)

S2 Fig Southern hybridization analysis for the PFGE gel. The *tet(M)* gene was used as a probe. The left two lanes are positive and negative controls for *tet*(M); the other 21 lanes represent the total DNA from each transconjugant. Sizes shown in left correspond to the positions of linear DNA included in ProMega-Markers Lambda Ladders in the ethidium bromide strained gel.
(PDF)

S3 Fig Consensus sequence found in the secondary structure alignment of MOB_H_ family relaxase. Secondary structure alignment was generated using PROMAL3D. The plasmid sequenced in this study (pSEA1) is highlighted in light glue. Consensus_ss: consensus secondary structure: h, alpha-helix; e, beta-sheet.
(PDF)

S4 Fig Long PCR analysis to assess the Tn*6283* integration pattern in transconjugants. (A) Four expected forms of a transconjugant chromosome carrying Tn*6283*. Form 3 and form 5 can be generated from form 1 via homologous recombination between Tn*6283* copies. Arrowheads indicate primer-annealing positions. Numbers in parentheses indicate the primer pair used for PCR and correspond to the lane number on the agarose gel images shown in panel B. (B) Results of long PCR. Pattern A suggests that form 2 and its derived form, form 3, are present in the cell population. Pattern B suggest that form 1, form 2, form 3, and form 5 are all present in the cell population. Fragments > 10 kb were detected in primer sets 5 and 6 in pattern B, which suggests the presence of a tandem repeat structure of Tn*6283* at *attL* and *attR* in a fraction of the cell population. Pattern C was obtained from strain LN15. In pattern C, intact *bcp* gene is present. About 7–8 kb PCR products in lane 4, 6, 7 (NS) are nonspecific target amplification caused by off-target annealing of primer 2599572R. 2 ul of PCR products were loaded in lane 2 and 3, while 1 ul of PCR products were loaded in other lanes to improve size recognition of the high molecular weight PCR products.
(PDF)

S5 Fig Excision of Tn*6283* in *Vibrio ponticus*. (A) Nucleotide sequences of Tn*6283*–pSEA1 borders. Expected nicking positions are indicated by arrowheads. (B) Similarity between the Tn*6283* insertion region in pSEA1 and a hypothetical protein gene in a contig derived from *V*. *nigripluchritudo* strain FTn2. (C) Design for PCR detection of recombination products. Black arrowheads indicate primers and their annealing positions. (D) PCR detection of joint formation on the recombination products. Primer sets used were 2F-2F2 for lane 1, 2F-1R3 for lane 2, and 3F-3R for lane 3. (E) Sequences of PCR-amplified joints on the circularized Tn*6283*. Four of the five clones sequenced were identical, which can be generated by strand exchange at two nicking sites indicated by blue arrowheads. One sequence can be generated by strand exchange at two nicking sites indicated by white arrowheads. Note that *V*. *ponticus* carries two Tn*6283* copies, one on pSEA1 and the other on the chromosome. (F) Sequences of PCR-amplified joints in the unoccupied Tn*6283* donor site. The cloned PCR products contained scar sequences, which can be generated by strand exchange at nicking sites indicated by blue and red arrowhead in panel A. (G) Strand exchange at the synaptic complex, potentially involved in generation of the scar sequence during incorrect excision.
(PDF)

S6 Fig Tn*6283* excision occurs independent of growth phase. Five overnight cultures were respectively diluted by ~1000-fold in 10 ml LB in L-shape tubes, and then incubated up to 4, 8, or 24 hr (corresponding to each growth phase) with agitation. Copy number of *att*_Tn*6283*_ and *dxs* (chromosome) were determined using qPCR as described in the Materials and Methods. D.L.: detection limit.
(PDF)

S7 Fig Stable maintenance of Tn*6283* in the *E*. *coli* cell population. In each assay, 40 colonies were investigated for the presence of a Tn*6283*-chromosome junction using PCR. Tn*6283*-free colonies were not detected throughout the experiment.
(PDF)
