# Supplementary material for: Interplay of a non-conjugative integrative element and a conjugative plasmid in the spread of antibiotic resistance via suicidal plasmid transfer from an aquaculture Vibrio isolate
Source: PLoS One. 2018 Jun 7;13(6):e0198613. doi: 10.1371/journal.pone.0198613 (PMC5991714; doi:10.1371/journal.pone.0198613)
Supplement: S1 Fig — One representative data of W3110 (A), LN3 (B), and LN5 (C). Number of fluorescence-signal positive dead cells in the boxed area was count and summarized in (D). (PDF) [file pone.0198613.s002.pdf]

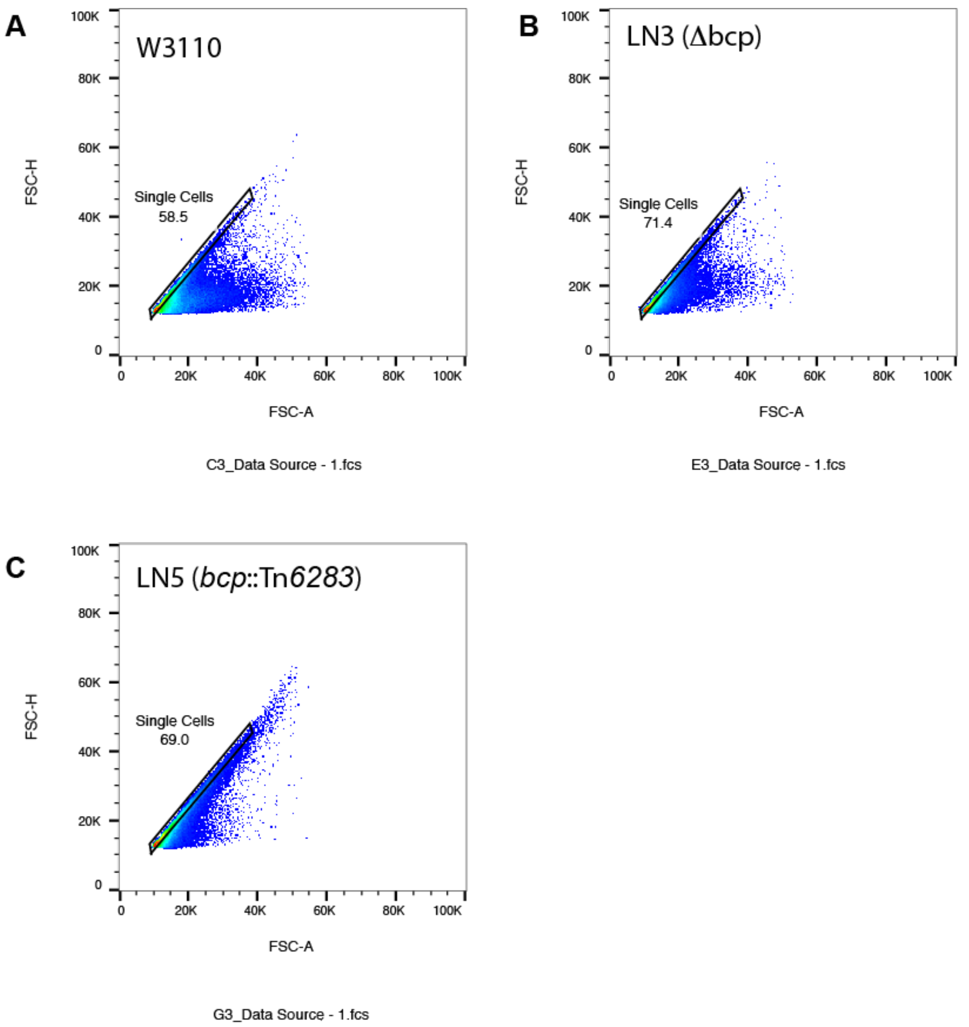

D

Sample_name	Strains	single cells	dead single cells	Fraction of dead cells
C2_Data Source - 1.fcs	W3110	55916	201	0.003581802
C3_Data Source - 1.fcs	W3110	55167	220	0.003972051
C4_Data Source - 1.fcs	W3110	56628	182	0.003203661
C5_Data Source - 1.fcs	W3110	56192	199	0.003528932
C6_Data Source - 1.fcs	W3110	55989	161	0.00286732
C7_Data Source - 1.fcs	W3110	57565	392	0.006763635
E2_Data Source - 1.fcs	delta_bcp	66205	77	0.001161703
E3_Data Source - 1.fcs	delta_bcp	68879	93	0.001348373
E4_Data Source - 1.fcs	delta_bcp	69949	85	0.001213696
E5_Data Source - 1.fcs	delta_bcp	69820	55	0.00078712
E6_Data Source - 1.fcs	delta_bcp	68577	66	0.000961496
E7_Data Source - 1.fcs	delta_bcp	69171	73	0.001054243
G2_Data Source - 1.fcs	LN5	64104	526	0.008138635
G3_Data Source - 1.fcs	LN5	66231	618	0.009244716
G4_Data Source - 1.fcs	LN5	66798	649	0.00962237
G5_Data Source - 1.fcs	LN5	68158	502	0.007311389
G6_Data Source - 1.fcs	LN5	66369	560	0.008367076
G7_Data Source - 1.fcs	LN5	65786	613	0.009232067

S1 Fig. Selection of single cells in flow cytometry analysis. (A) one representative data of W3110. (B) LN3. (C) LN5. Number of fluorescence-signal positive dead cells in the boxed area was count and summarized in (D).
