# Supplementary material for: Interplay of a non-conjugative integrative element and a conjugative plasmid in the spread of antibiotic resistance via suicidal plasmid transfer from an aquaculture Vibrio isolate
Source: PLoS One. 2018 Jun 7;13(6):e0198613. doi: 10.1371/journal.pone.0198613 (PMC5991714; doi:10.1371/journal.pone.0198613)
Supplement: S2 Fig — The tet(M) gene was used as a probe. The left two lanes are positive and negative controls for tet(M); the other 21 lanes represent the total DNA from each transconjugant. Sizes shown in left correspond to the positions of linear DNA included in ProMega-Markers Lambda Ladders in the ethidium bromide strained gel. (PDF) [file pone.0198613.s003.pdf]

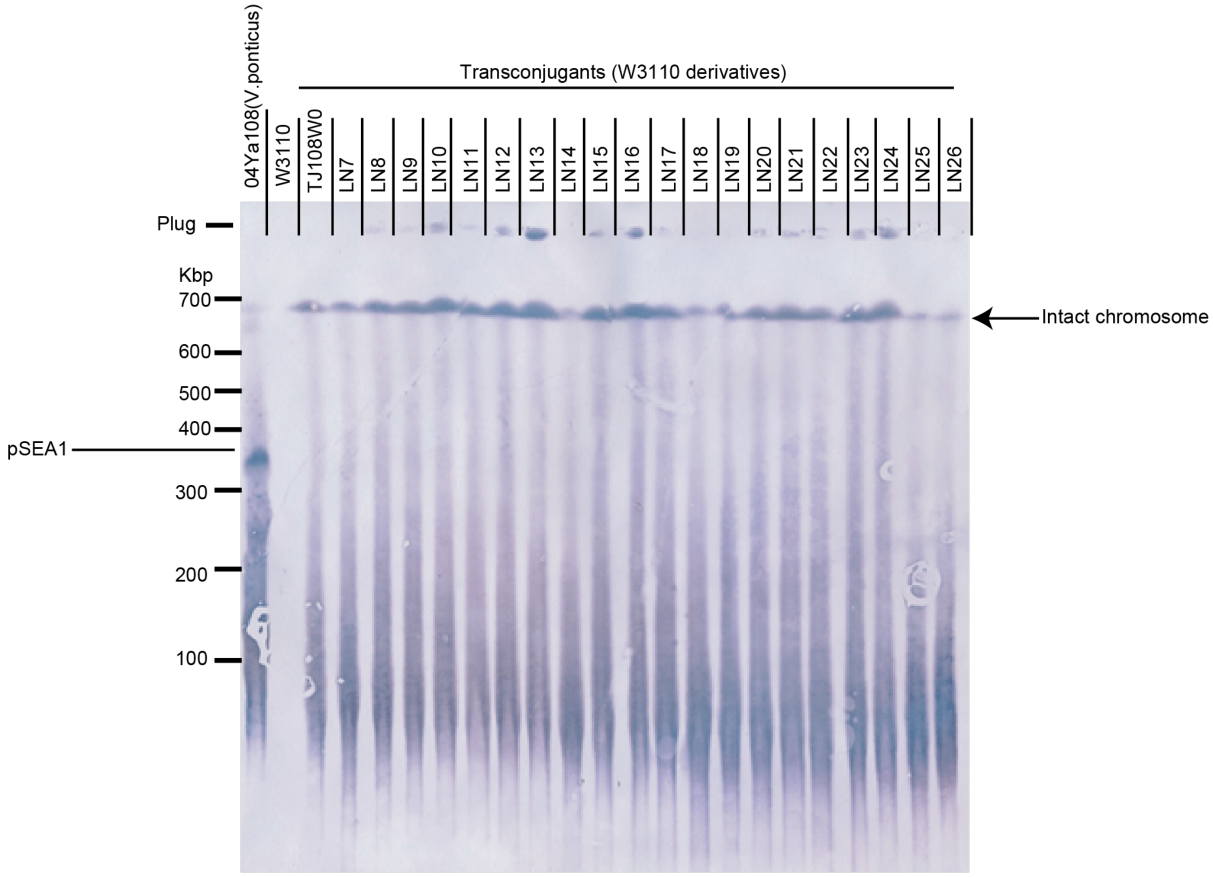

S2 Fig. Southern hybridization analysis for the PFGE gel. The *tet(M)* gene was used as a probe. The left two lanes are positive and negative controls for *tet*(M); the other 21 lanes represent the total DNA from each transconjugant. Sizes shown in left correspond to the positions of linear size markers included in ProMega-Markers Lambda Ladders in the ethidium bromide strained gel.
