# Supplementary material for: Interplay of a non-conjugative integrative element and a conjugative plasmid in the spread of antibiotic resistance via suicidal plasmid transfer from an aquaculture Vibrio isolate
Source: PLoS One. 2018 Jun 7;13(6):e0198613. doi: 10.1371/journal.pone.0198613 (PMC5991714; doi:10.1371/journal.pone.0198613)
Supplement: S4 Fig — (A) Four expected forms of a transconjugant chromosome carrying Tn6283. Form 3 and form 5 can be generated from form 1 via homologous recombination between Tn6283 copies. Arrowheads indicate primer-annealing positions. Numbers in parentheses indicate the primer pair used for PCR and correspond to the lane number on the agarose gel images shown in panel B. (B) Results of long PCR. Pattern A suggests that form 2 and its derived form, form 3, are present in the cell population. Pattern B suggest that form 1, form 2, form 3, and form 5 are all present in the cell population. Fragments > 10 kb were detected in primer sets 5 and 6 in pattern B, which suggests the presence of a tandem repeat structure of Tn6283 at attL and attR in a fraction of the cell population. Pattern C was obtained from strain LN15. In pattern C, intact bcp gene is present. About 7–8 kb PCR products in lane 4, 6, 7 (NS) are nonspecific target amplification caused by off-target annealing of primer 2599572R. 2 ul of PCR products were loaded in lane 2 and 3, while 1 ul of PCR products were loaded in other lanes to improve size recognition of the high molecular weight PCR products. (PDF) [file pone.0198613.s005.pdf]

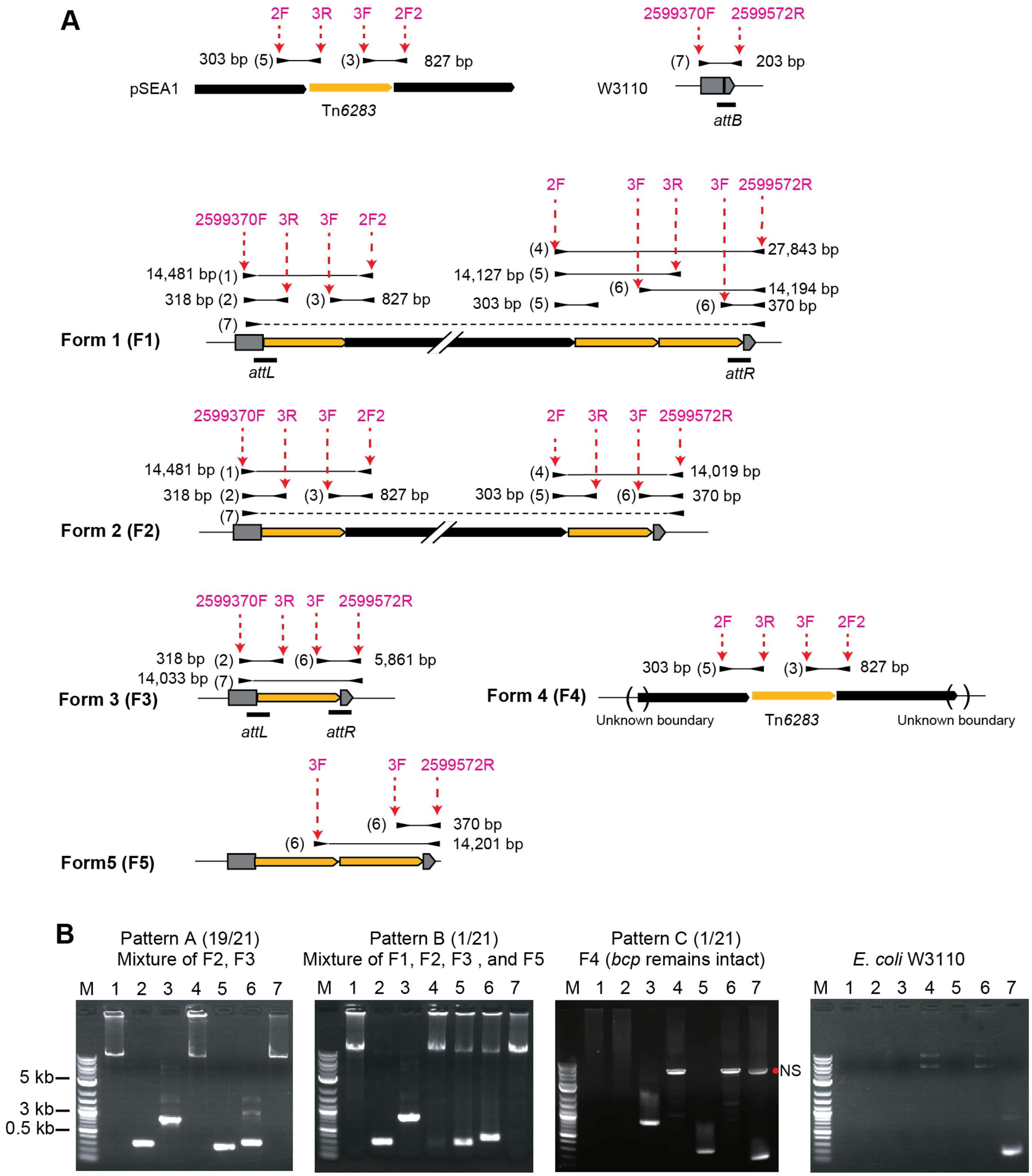

S4 Fig. Long PCR analysis to assess the Tn*6283* integration pattern in transconjugants. (A) Four expected forms of a transconjugant chromosome carrying Tn*6283*. Form 3 and form 5 can be generated from form 1 via homologous recombination between Tn*6283* copies. Arrowheads indicate primer-annealing positions. Numbers in parentheses indicate the primer pair used for PCR and correspond to the lane number on the agarose gel images shown in panel B. (B) Results of long PCR. 2 ul of PCR products was loaded in lane 2 and 3, while 1 ul of PCR products was loaded in other lanes to improve size recognition of the high molecular weight PCR products. Pattern A suggests that form 2 and its derived form, form 3, are present in the cell population. Pattern B suggest that form 1, form 2, form 3, and form 5 are all present in the cell population. Fragments > 10 kb were detected in primer sets 5 and 6 in pattern B, which suggests the presence of a tandem repeat structure of Tn*6283* at *attL* and *attR* in a fraction of the cell population. Pattern C was obtained from strain LN15. In pattern C, the intact *bcp* gene is present. About 7-8 kb PCR products in lane 4, 6, 7 (NS) are nonspecific target amplification caused by off-target annealing of primer 2599572R.
