# Supplementary material for: Interplay of a non-conjugative integrative element and a conjugative plasmid in the spread of antibiotic resistance via suicidal plasmid transfer from an aquaculture Vibrio isolate
Source: PLoS One. 2018 Jun 7;13(6):e0198613. doi: 10.1371/journal.pone.0198613 (PMC5991714; doi:10.1371/journal.pone.0198613)
Supplement: S5 Fig — (A) Nucleotide sequences of Tn6283–pSEA1 borders. Expected nicking positions are indicated by arrowheads. (B) Similarity between the Tn6283 insertion region in pSEA1 and a hypothetical protein gene in a contig derived from V. nigripluchritudo strain FTn2. (C) Design for PCR detection of recombination products. Black arrowheads indicate primers and their annealing positions. (D) PCR detection of joint formation on the recombination products. Primer sets used were 2F-2F2 for lane 1, 2F-1R3 for lane 2, and 3F-3R for lane 3. (E) Sequences of PCR-amplified joints on the circularized Tn6283. Four of the five clones sequenced were identical, which can be generated by strand exchange at two nicking sites indicated by blue arrowheads. One sequence can be generated by strand exchange at two nicking sites indicated by white arrowheads. Note that V. ponticus carries two Tn6283 copies, one on pSEA1 and the other on the chromosome. (F) Sequences of PCR-amplified joints in the unoccupied Tn6283 donor site. The cloned PCR products contained scar sequences, which can be generated by strand exchange at nicking sites indicated by blue and red arrowhead in panel A. (G) Strand exchange at the synaptic complex, potentially involved in generation of the scar sequence during incorrect excision. (PDF) [file pone.0198613.s006.pdf]

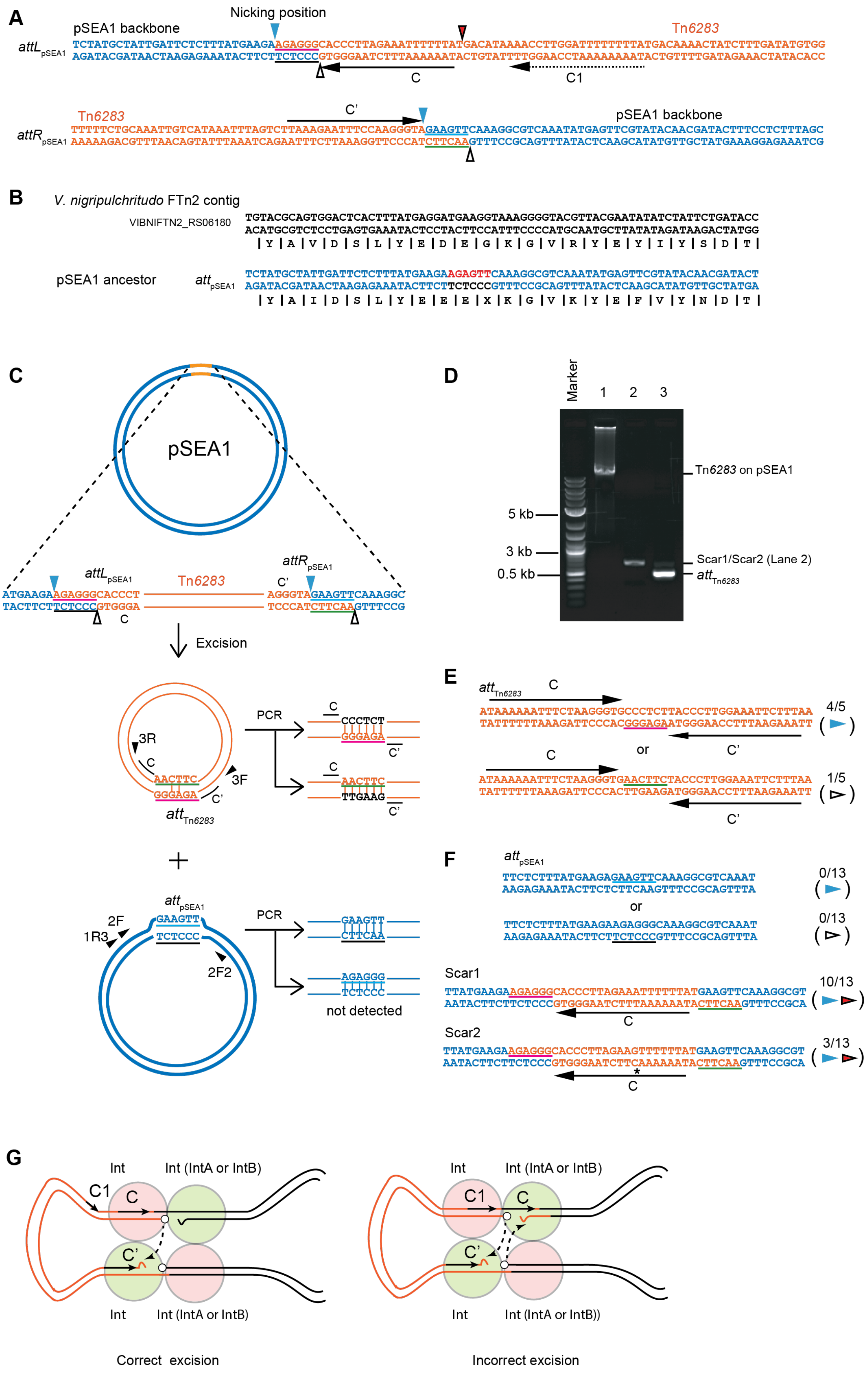

A
Nicking position
pSEA1 backbone
Tn6283
attL pSEA1
C
C1
Tn6283
C'
pSEA1 backbone
attR pSEA1
B
V. nigripulchritudo FTn2 contig
VIBNIFTN2_RS06180
pSEA1 ancestor
att pSEA1
C
pSEA1
attL pSEA1
Tn6283
attR pSEA1
C'
C
Excision
PCR
3R
3F
att Tn6283
2F
1R3
2F2
att pSEA1
not detected
D
Marker
1
2
3
Tn6283 on pSEA1
5 kb
3 kb
0.5 kb
Scar1/Scar2 (Lane 2)
att Tn6283
E
att Tn6283
4/5
or
1/5
F
att pSEA1
0/13
or
0/13
Scar1
10/13
Scar2
3/13
G
Int
Int (IntA or IntB)
C1
C
C'
Int
Int (IntA or IntB)
Correct excision
Int
Int (IntA or IntB)
C1
C
C'
Int
Int (IntA or IntB))
Incorrect excision

S5 Fig. Excision of Tn*6283* in *Vibrio ponticus*. (A) Nucleotide sequences of Tn*6283*–pSEA1 borders. Expected nicking positions are indicated by arrowheads. (B) Similarity between the Tn*6283* insertion region in pSEA1 and a hypothetical protein gene in a contig derived from *V. nigripluchritudo* strain FTn2. (C) Design for PCR detection of recombination products. Black arrowheads indicate primers and their annealing positions. (D) PCR detection of joint formation on the recombination products. Primer sets used were 2F-2F2 for lane 1, 2F-1R3 for lane 2, and 3F-3R for lane 3. (E) Sequences of PCR-amplified joints on the circularized Tn*6283.* Four of the five clones sequenced were identical, which can be generated by strand exchange at two nicking sites indicated by blue arrowheads. One sequence can be generated by strand exchange at two nicking sites indicated by white arrowheads. Note that *V. ponticus* carries two Tn*6283* copies, one on pSEA1 and the other on the chromosome. (F) Sequences of PCR-amplified joints in the unoccupied Tn*6283* donor site. The cloned PCR products contained scar sequences, which can be generated by strand exchange at nicking sites indicated by blue and red arrowhead in panel A. (G) Strand exchange at the synaptic complex, potentially involved in generation of the scar sequence during incorrect excision.
