# Supplementary material for: Interplay of a non-conjugative integrative element and a conjugative plasmid in the spread of antibiotic resistance via suicidal plasmid transfer from an aquaculture Vibrio isolate
Source: PLoS One. 2018 Jun 7;13(6):e0198613. doi: 10.1371/journal.pone.0198613 (PMC5991714; doi:10.1371/journal.pone.0198613)
Supplement: S6 Fig — Five overnight cultures were respectively diluted by ~1000-fold in 10 ml LB in L-shape tubes, and then incubated up to 4, 8, or 24 hr (corresponding to each growth phase) with agitation. Copy number of attTn6283 and dxs (chromosome) were determined using qPCR as described in the Materials and Methods. D.L.: detection limit. (PDF) [file pone.0198613.s007.pdf]

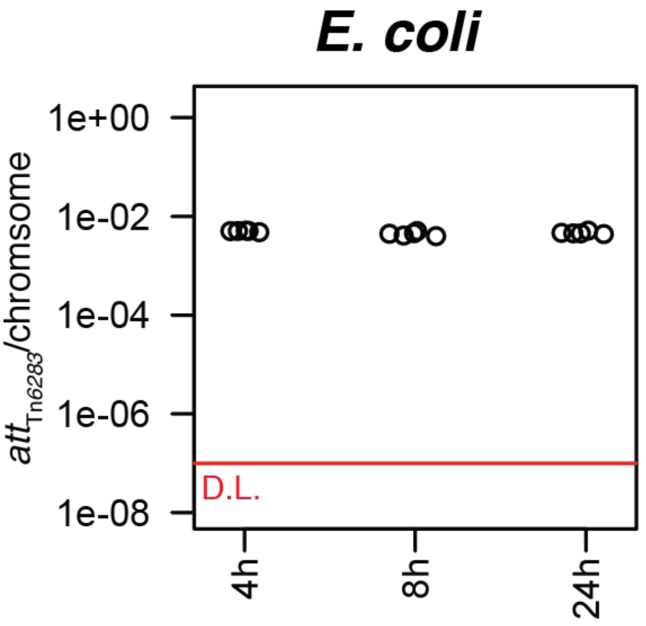

S6 Fig. Tn*6283* excision occurs independent of growth phase. Five overnight cultures were respectively diluted by ~1000-fold in 50 ml LB in flasks, and then incubated up to 4, 8, or 24 hr (corresponding to each growth phase) with agitation. Copy number of att_{Tn6283} and *dxs* (chromosome) were determined using qPCR as described in the Materials and Methods. D.L.: detection limit.
