# Supplementary material for: Interplay of a non-conjugative integrative element and a conjugative plasmid in the spread of antibiotic resistance via suicidal plasmid transfer from an aquaculture Vibrio isolate
Source: PLoS One. 2018 Jun 7;13(6):e0198613. doi: 10.1371/journal.pone.0198613 (PMC5991714; doi:10.1371/journal.pone.0198613)
Supplement: S7 Fig — In each assay, 40 colonies were investigated for the presence of a Tn6283-chromosome junction using PCR. Tn6283-free colonies were not detected throughout the experiment. (PDF) [file pone.0198613.s008.pdf]

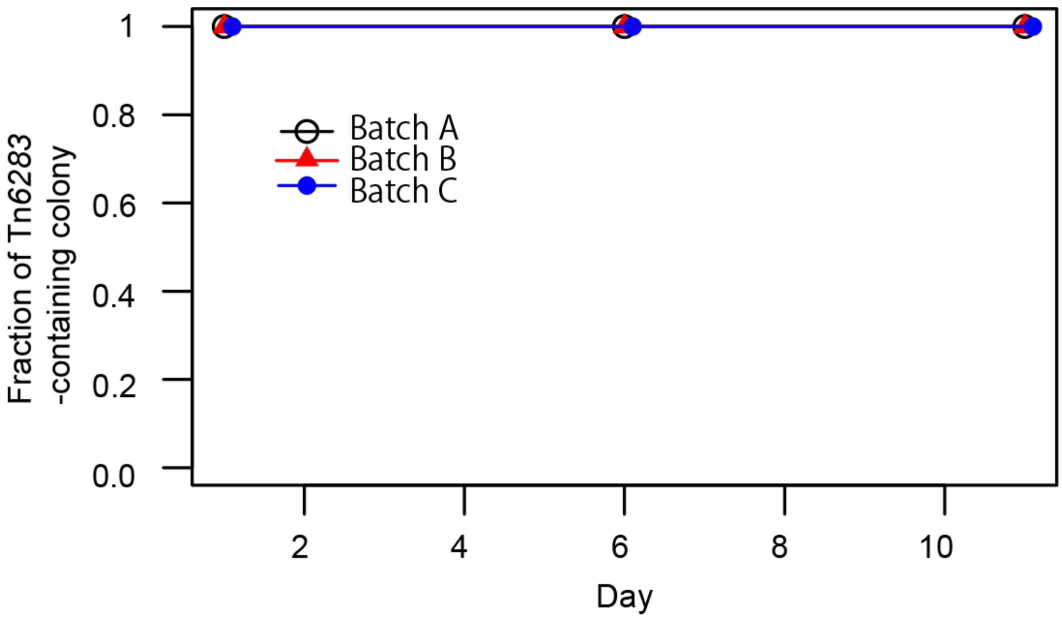

S7 FIG. Stable maintenance of Tn*6283* in the *E. coli* cell population. In each assay, 40 colonies were investigated for the presence of a Tn*6283*–chromosome junction using PCR. Tn*6283*-free colonies were not detected throughout the experiment.
