# Supplementary material for: Interplay of a non-conjugative integrative element and a conjugative plasmid in the spread of antibiotic resistance via suicidal plasmid transfer from an aquaculture Vibrio isolate
Source: PLoS One. 2018 Jun 7;13(6):e0198613. doi: 10.1371/journal.pone.0198613 (PMC5991714; doi:10.1371/journal.pone.0198613)
Supplement: S3 Fig — Secondary structure alignment was generated using PROMAL3D. The plasmid sequenced in this study (pSEA1) is highlighted in light glue. Consensus_ss: consensus secondary structure: h, alpha-helix; e, beta-sheet. (PDF) [file pone.0198613.s004.pdf]

```
pSEA1        : 71 DKEFDRIIRPIIHHVIRYFMSLPASERHHHTDMSASLFYHSLQTANIALAKYNS 124
ICE_Vnig     : 71 DEQFNLIIRPILHHVIKYFMALPASERHHHTDMSASLFFHSLQTANIALAKYNS 124
R391         : 60 QRDFQRLIQPCIDHLAAYVHLLPASEHHHHSG-AGGLLRHSLEVAFWAAQAAEG 112
SXT          : 60 QREFQRLIQPCIDHLAAYVHLLPASEHHHHSG-AGGLLRHSLEVAFWAAQAAEG 112
pAsa4        : 65 IEDFNRLVLPVIHRYAAFVHLLPASEAHHHRG-AGGLFRHGLEVAFWAAQASES 117
pIP1202      : 72 VDDFNRLVLPVIQRYAAFVHLLPASESHHHRG-AGGLFRHGLEVAFWAAQASES 124
pAQU1        : 70 QEEYDRLVMPVIGRYAAFVHLLPASEAHHHRG-AGGLFRHGLEVAFWATQASES 122
Pls_Rhofe    : 85 NDDFERRYLAPLRRAAELINLIPATRDKHHTG-AGGLFRFAATMAIRSAQSADG 137
Pls_Shesp    : 99 VYNAQTLVRDVVANFLSYAHLLPASENHHHAA-VGGLARHSLEVALFSLRHMAS 151
Chr_Neigo    : 79 DENWSKDALPFLEKYIAFVQRLPASESHHHAG-DGGLVRHTLDVAALALVASTS 131
pKLC102      : 55 KQQFEEIYRRPLANYAELVQQLPASENHHHAH-PGGMIDHGLEIVAYALKVRQT 107
PAGI-4_C     : 24 KQQFEEIYRRPLANYAELVQQLPASENHHHAH-PGGMIDHGLEIVAYALKVRQT  76
clc          : 58 RKQFATLYRAPLERYAELVQRFPASEAHHHAY-PGGMLDHGLEIVAYALKLRQS 110
PAGI-3SG     : 58 RQQFTALYLAPLERYASLVQQFPASESHHHAY-SGGMLDHGLEIVAYALKLRQS 110
PAGI-2_C     : 58 RKQFAVLYRAPLERYAELVQAFPASESHHHAY-PGGMLDHGLEIVAYALKLRQS 110
pCAR1        :139 ASDLKLYVEPILVNFAELAHMLPASENHHHSG-PGGLLRHSLEVASLTLDGCLT 191
R478         :106 AYTFTNLVLKPLVEYVRWIHLLPASENHHHNG-IGGLLSHSLEVAILSLKNAHH 158
R27          :104 AYTFTNLVLKPLIEYIRWIHLLPASENHHHNG-IGGLLSHSLEVAMISLKNANH 156
Rts1         : 76 DAEYDEYIQPMLTNFANFVHLLPASEFHHHRA-QGGLLRHTLEVVLYSIKIAKS 128
pMAQU02      : 51 EQVWNKHYLYAIEQFAELVQEVPASEIHHHSE-SGGLIDHTLEALYAGVRISQG 103
Consensus_aa                            PASE HHH    GGL  H L
Consensus_ss      hhhhhhhhhhhhhhhhhhhhh              hhhhhhhhhhhhhhhhh
```

```
pSEA1        :132 IKIEHKNEYKHAAGVAVFLAALFHDAGKLFS-DMSVYPSDEK 172
ICE_Vnig     :132 IKIEHKNEYKHAASVAVFLAALFHDAGKLYS-DMSVYPSDEK 172
R391         :119 GTPVEKKELEPRWRVAAALGGLFHDIGKPVS-DLSITDEDGR 159
SXT          :119 GTPVEKKELEPRWRVAAALGGLFHDIGKPVS-DLSITDEDGR 159
pAsa4        :124 GTPKQRRDNEPRWRLASCFSGLLHDVGKPLA-DVSITDKNGV 164
pIP1202      :131 GTPRERRDNEPRWRLASCFSGLLHDVGKPLS-DVSITDKDGS 171
pAQU1        :129 GSPRERRNNEPRWRLACCFSGLLHDVGKPLS-DVVITNSDGS 169
Pls_Rhofe    :144 EGIERRRQTEAAWRHAAFLTGLTCELFRPLT-EMMIFDPKGN 184
Pls_Shesp    :158 KFTDELHGKKIRWQYAAWVIGLVHDIGKALH-DMTVQADDS- 197
Chr_Neigo    :138 AKTEEIAKKTAVWRYGIMCAALLHDVGKTVT-GFQVELFDSA 178
pKLC102      :114 AAPESQSAQTEAWSAAAAYGALAHDLGKIVV-DLQVELQDGS 154
PAGI-4_C     : 83 AAPESQSAQAEAWSAAAAYGALAHDIGKIVV-DLQVELQDGS 123
clc          :117 STPEDQAAQSEAWTAAVAYAALLHDVGKLAV-DLHVELADGS 157
PAGI-3SG     :117 TTPEDQVIQAEAWTAAIAYAALLHDIGKVAV-DLHVEYGDGS 157
PAGI-2_C     :117 ASPEDQAAQAEAWTAAVAYAALLHDIGKIAV-DLHVELADGS 157
pCAR1        :198 ETPARRSMRLRRWYVAGIASGLLHDAGKPLT-DIRATDFEGN 238
R478         :165 GYQDEEVVRRKVYLYAAFICGLVHDAGKVYDLDIVSLNLASP 206
R27          :163 GYQDEEVVRRKVYLYAAFICGLVHDAGKVYDLDIVSLNLSET 204
Rts1         :135 ESPVIKSDRALAWRIAVVVGAVMHDIGKPIS-DVDVWDKSGE 175
pMAQU02      :110 AEPESIAESADRWRFAAMIAILSHDLGKIVT-DIEVVYRLPE 150
Consensus_aa                           L HD GK     D
Consensus_ss      hhhhhhhhhhhhhhhhhhhhhhh  eeee eeeeeee
```

S3 Fig. Consensus sequence found in the secondary structure alignment of MOB_H family relaxase. Secondary structure alignment was generated using PROMAL3D. The plasmid sequenced in this study (pSEA1) is highlighted in light blue. Consensus_ss: consensus secondary structure: h, alpha-helix; e, beta-sheet.
